# Isolation and Characterization of Phenylpropanoid and Lignan Compounds from *Peperomia pellucida* [L.] Kunth with Estrogenic Activities

**DOI:** 10.3390/molecules25214914

**Published:** 2020-10-23

**Authors:** I Gusti Agung Ayu Kartika, In Jae Bang, Catur Riani, Muhamad Insanu, Jong Hwan Kwak, Kyu Hyuck Chung, I Ketut Adnyana

**Affiliations:** 1 Pharmacology and Clinical Pharmacy Department, School of Pharmacy, Institut Teknologi Bandung, Ganesha 10, Bandung 40132, Indonesia; kartikaayu269@students.itb.ac.id; 2 Prevent Pharm Laboratory, School of Pharmacy, Sungkyunkwan University, Suwon-Si, Gyeonggi-Do 16419, Korea; injae753@naver.com; 3 Laboratory of Pharmaceutical Biotechnology, School of Pharmacy, Institut Teknologi Bandung, Ganesha 10, Bandung 40132, Indonesia; catur@fa.itb.ac.id; 4 Pharmaceutical Biology Department, School of Pharmacy, Institut Teknologi Bandung, Ganesha 10, Bandung 40132, Indonesia; insanu@fa.itb.ac.id; 5 Phytochemistry Laboratory, School of Pharmacy, Sungkyunkwan University, Suwon-Si, Gyeonggi-Do 16419, Korea

**Keywords:** *Peperomia pellucida*, phenylpropanoid and lignan, phytoestrogen, E-SCREEN, docking

## Abstract

Extracts of *Peperomia pellucida* [L.] Kunth have previously been demonstrated to have in vivo estrogenic-like effects, thereby functioning as an anti-osteoporotic agent. However, the compounds responsible for these effects have not yet been determined. Therefore, the aim of this study is to isolate and elucidate potential compounds with estrogenic activity. The structures of the isolated compounds were identified using 1D ^1^H and ^13^C-NMR and confirmed by 2D FT-NMR. The estrogenic activity was evaluated using the E-SCREEN assay, and a molecular docking study was performed to predict the binding affinity of the isolated compounds to estrogen receptors. In this experiment, we successfully isolated three phenylpropanoids and two lignan derivatives, namely, 6-allyl-5-methoxy-1,3-benzodioxol-4-ol (**1**), pachypostaudin B (**2**), pellucidin A (**3**), dillapiole (**4**), and apiol (**5**). Among these compounds, the isolation of **1** and **2** from *P. pellucida* is reported for the first time in this study. Activity assays clearly showed that the ethyl acetate extract and its fractions, subfractions, and isolated compounds exerted estrogenic activity. Methanol fraction of the ethyl acetate extract produced the highest estrogenic activity, while **1** and **2** had partial agonist activity. Some compounds (derivates of dillapiole and pellucidin A) also had, in addition, anti-estrogenic activity. In the docking study, the estrogenic activities of **1**–**5** appeared to be mediated by a classical ligand-dependent mechanism as suggested by the binding interaction between the compounds and estrogen receptors; binding occurred on Arg 394 and His 524 of the alpha receptor and Arg 346 and His 475 of the beta receptor. In summary, we reveal that *P. pellucida* is a promising anti-osteoporotic agent due to its estrogenic activity, and the compounds responsible for this activity were found to be lignan and phenylpropanoid derivatives. The presence of other compounds in either the extract or fraction may contribute to a synergistic effect, as suggested by the higher estrogenic activity of the methanol fraction. Hence, we suggest further research on the osteoporotic activity and safety of the identified compounds, especially regarding their effects on estrogen-responsive organs.

## 1. Introduction

Estrogens are well-known steroid hormones. Estrogens act on various tissues and are involved in numerous physiological processes. They regulate the establishment of reproductive organs in both females and males, the protection and differentiation of the central nervous system, bone remodeling and growth, lipid metabolism in the liver, and vasodilation of the cardiovascular system [1,2]. The activity of estrogen is mainly mediated by two different estrogen receptors, ERα and Erβ, which work antagonistically [3,4].

A range of both natural and synthetic ligands can modulate estrogenic activity. Some of these are referred to as selective ER modulators (SERMs) because they can act like estrogens in some tissues but block estrogen action in others, thus exerting tissue-specific effects by acting on selective ERs [5]. Accordingly, phytoestrogens, a large group of plant-derived compounds that have estrogenic properties, can also act as SERMs [5,6]. Phytoestrogens have varying degrees of estrogenic activity, and their interactions with the estrogen receptor are in some ways similar to the SERM/ER interaction [7]. This kind of activity offers pharmacological or nutraceutical advantages [8,9,10]. The groups of phenolic compounds that are currently classified as phytoestrogens include the chalcones, flavonoids (isoflavonoids, flavones, flavonols, flavanones), stilbenoids, and lignans [6,8,9,10,11].

*Peperomia pellucida* is a plant that is used worldwide as both a medicinal plant and vegetable. Application of its ethanol and aqueous extracts has shown typical estrogenic-like effects in in vivo anti-osteoporosis tests, including increased uterine weight and preventing bone loss in ovariectomized rats, and promoting fracture healing in drill hole injuries in rats, which are commonly used as an animal model in osteoporosis studies [12,13,14]. Estrogens are known to have direct effects on osteoclasts, osteocytes, and osteoblasts, leading to decreased bone resorption, inhibition of bone remodeling, and maintenance of bone formation, respectively [15]. Recently, we demonstrated that an ethyl acetate extract from this plant had a partial agonist effect in estrogenic tests using the MCF-7/BUS cell line. However, there are no data showing which compounds contribute to this estrogenic effect. Therefore, it is important to identify and characterize these compounds to understand the mechanisms underlying their estrogenic effect. Since phytoestrogens can contribute to the bone healing process, such compounds in *P. pellucida* are conceivably potential candidates for anti-osteoporotic agents. In this study, we aimed to isolate compounds from the ethyl acetate extract of *P. pellucida* potentially responsible for its estrogenic activity and conduct estrogenic tests on the compounds along with the extract, fractions, and subfractions.

It is important to determine the estrogenic and anti-estrogenic activities of the samples to evaluate their potential clinical applications and more clearly identify the responsible compounds. Estrogenic activity is related to anti-osteoporotic effects, while anti-estrogenic activity is associated with anti-cancer properties. This knowledge is also important in predicting the safety of the samples, especially in the context of cancer and other diseases that can be influenced by estrogen [16].

This study was carried out using MCF-7/BUS cells—estrogen receptor-positive breast cancer cells. Besides the expression of the progesterone receptor and human epidermal growth factor receptor 2 (HER2), these cells also express receptors for estrogen, such as ERα, ERβ, and G-protein coupled receptor 30 [17,18]. Full-length ERα and ERβ are reported to be expressed in various tissues. Estrogen receptors (ERs) have been found to localize to the nucleus, mitochondria, and plasma membrane (around 5%–10% of the population) [19]. ERs have also been found in discrete cytoplasmic organelles, such as the endoplasmic reticulum [20]. In breast cancer cells, the plasma membrane mainly contains ERα, with scant ERβ present [21]. ERα is present in approximately 75% of breast cancers [22].

The biological activities of estrogens are mediated by both genomic and non-genomic pathways [23,24,25]. Genomic effects are those involving the migration of estrogen–receptor complexes to the cell nucleus and direct interaction with chromatin at specific DNA sequences referred to as estrogen response elements (EREs). In direct genomic signaling (classical mechanism of estrogen signaling), the nuclear estrogen receptors ERα and ERβ act as ligand-activated transcription factors, whereas in indirect genomic signaling, the estrogen receptor complexes act through protein–protein interactions with other transcription factors and response elements. On the other hand, non-genomic effects involve indirect regulation of gene expression through a variety of intracellular signaling events. In the non-genomic pathway, E_2_ binds to ERs localized in the cell membrane and activates signal transduction pathways or functions via activation of the GPR30 signaling cascade, thus stimulating PI3K/Akt and MAPK (mitogen-activated protein kinase) signaling intermediates [23,24,25]. The E-SCREEN assay and molecular docking studies were used to assess the estrogenic activity of samples and determined the mechanism for this through the characterization of the interaction with ERs.

## 2. Results

### 2.1. Isolation and Identification of Compounds from P. pellucida Ethyl Acetate Extract

**Figure 1:**
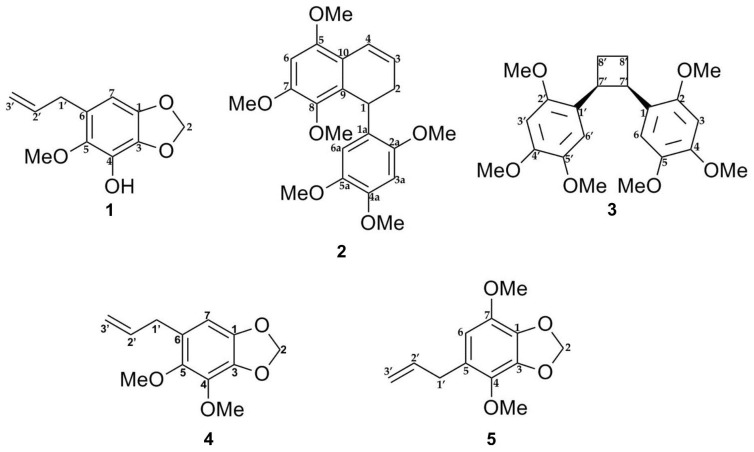
Chemical structures of compounds **1**–**5** isolated from the aerial part of *P. pellucida* as described in materials and methods.

The E-SCREEN bioassay for assessing estrogenic activity was used to guide the isolation of five compounds from the ethyl acetate extract of *P. pellucida*. The ethyl acetate extract of the aerial parts of *P. pellucida* was further extracted by suspending in methanol followed by partitioning with petroleum ether. The methanol fraction, which demonstrated more potent estrogenic activity, was fractionated by silica gel chromatography. Selected fractions were rechromatographed on silica gel, RP-C18 columns, Sephadex LH-20 columns, Lobar columns, and recycling preparative high-performance liquid chromatography (HPLC) with a JAIGEL-GS310 column using different solvent combinations. All of the isolated compounds were classified as lignans and phenylpropanoids. The structures of the compounds are shown in Figure 1.

Lignans are natural products characterized by a phenylpropanoid core (C_6_C_3_ unit). Structurally, they are composed of two or more phenylpropanoid units as a basic scaffold [26,27,28]. Compounds **1**, **4**, and **5** are classified as phenylpropanoids, which contain a planar methylenedioxyphenyl moiety with allyl group [28]. Compounds **2** and **3** are classified as bisnorlignans. Norlignans are natural compounds based on diphenylpentanes, derived by the union of two phenylpropanoid units in the positions α, β′ or β, γ′ and characterized by the loss of the terminal carbon of the chain [29,30,31]. In bisnorlignans, this loss forces the chain to be arranged differently from in lignans (the junction between two phenylpropanoid units is through a β–β (8–8′) bond) and *neo*-lignans (the junction is not β–β type), where chirality plays a central role [32].

The structures of the compounds were elucidated by NMR spectral analysis and were in agreement with those reported in the literature [33,34].

6-Allyl-5-methoxy-1,3-benzodioxol-4-ol (**1**)

^1^H-NMR (400 MHz, CDCl_3_): δ 5.91 (2H, *s,* H-2), 6.27 (1H, *s*, H-7), 3.33 (2H, *d*, 6.44 Hz, H-1′), 5.90 (1H, *m,* H-2′), 5.07 (1H, *m,* H-3′a), 5.06 (1H, *m,* H-3′b), 3.76 (3H, *s*, 5-OMe), 5.36 (1H, *brs*, OH).

^13^C-NMR (213.8 MHz, CDCl_3_): δ 145.2 (C-1), 101.9 (C-2), 132.8 (C-3), 133.6 (C-4), 141.2 (C-5), 125.3 (C-6), 101.2 (C-7), 33.8 (C-1′), 137.2 (C-2′), 116.2 (C-3″), 62.1 (5-OMe).

1,1′-(1R,2S)-Cyclobutane-1,2-diylbis(2,4,5-trimethoxybenzene) (**3**, Pellucidin A)

^1^H-NMR (400 MHz, CDCl_3_): δ 6.48 (2H, *s*, H-3/3′ ), 6.98 (2H, *s*, H-6/6′), 3.85 (2H, *m*, H-7/7′), 2.29 (2H, *m*, H-8/8′α), 1.92 (2H, *m*, H-8/8′β), 3.75 (6H, *s*, 4/4′-OMe), 3.85 and 3.86 (each 6H, *s*, 2/2′-OMe and 5/5′-OMe).

^13^C-NMR (213.8 MHz, CDCl_3_): δ 124.8 (C-1/1′), 147.8 (C-2/2′), 98.0 (C-3/3′), 151.3 (C-4/4′), 143.3 (C-5/5′), 112.0 (C-6/6′), 40.7 (C-7/7′), 27.3 (C-8/8′), 56.5 (4/4′-OMe), 56.8 and 56.8 (2/2′-OMe and 5/5′-OMe).

4,5-Dimethoxy-6-prop-2-enyl-1,3-benzodioxole (**4**, Dillapiole)

^1^H-NMR (400 MHz, CDCl_3_): δ 5.88 (2H, *s,* H-2), 6.35 (1H, *s*, H-7), 3.30 (2H, *d*, 6.53 Hz, H-1′), 5.93 (1H, *m,* H-2′), 4.99 (2H, *m,* H-3′), 4.01 (3H, *s*, 4-OMe), 3.75 (3H, *s*, 5-OMe).

^13^C-NMR (100 MHz, CDCl_3_): δ 144.6 (C-1), 101.1 (C-2), 136.0 (C-3), 137.6 (C-4), 144.3 (C-5), 126.1 (C-6), 102.8 (C-7), 33.9 (C-1′), 137.4 (C-2′), 115.6 (C-3′), 60.0 (4-OMe), 61.3 (5-OMe).

4,7-Dimethoxy-5-prop-2-enyl-1,3-benzodioxole (**5**, Apiol)

^1^H-NMR (400 MHz, CDCl_3_): δ 5.95 (2H, *s,* H-2), 6.30 (1H, *s*, H-6), 3.31 (2H, *d*, 6.48 Hz, H-1′), 5.94 (1H, *m,* H-2′), 5.04 (2H, *m,* H-3′), 3.88 (3H, *s*, 4-OMe), 3.85 (1H, *s*, 7-OMe).

^13^C-NMR (213.8 MHz, CDCl_3_): δ 135.3 (C-1), 101.8 (C-2), 139.0 (C-3), 136.5 (C-4), 126.0 (C-5), 108.4 (C-6), 139.3 (C-7), 34.3 (C-1′), 137.6 (C-2′), 115.6 (C-3′), 60.4 (4-OMe), 57.1 (7-OMe).

**Figure 2:**
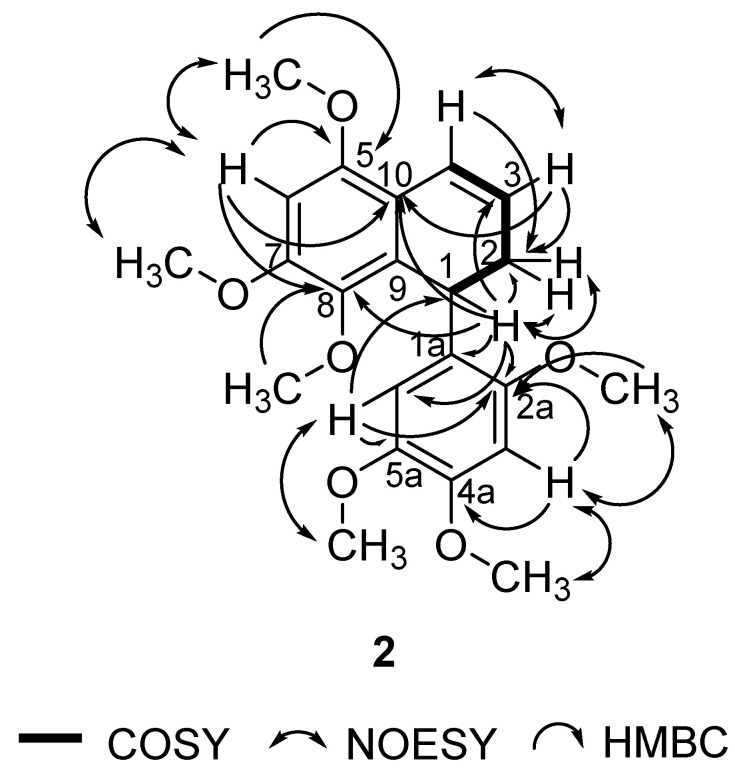
Key COSY, NOESY, and HMBC Correlations of Pachypostaudin B (**2**).

In this study, **1** was isolated for the first time from a natural source, and **2** was isolated for the first time from *P. pellucida*. The spectra of **1** were compared with the literature values of **4 [34]** since they have similar structures, with the exception of a different group attached to C-4. In the 6-OCH_3_ position, **4** has a methoxyl group whereas **1** has a hydroxyl group. 2D NMR analysis was conducted to confirm the chemical structure of **2**. ^1^H, ^13^C, DEPT 45°, DEPT 90°, DEPT 135°, COSY, NOESY, HSQC, and HMBC NMR data are shown in Table 1, while COSY, NOESY, and HMBC correlations are shown in Figure 2. The ^1^H and ^13^C data of **2** are in agreement with the literature, which confirmed that **2** was a 9,9′-bisnorlignan, 5,7,8-trimethoxy-1-(2,4,5-trimethoxyphenyl)-1,2-dihydronaphthalene or pachypostaudin B [35,36].

### 2.2. Cytotoxicity Assay

Cytotoxicity assays were conducted prior to estrogenic tests. For this, we used the WST-1 method. Cell viability (in %) in the presence of fractions, subfractions, and five isolated compounds from the ethyl acetate extract of *P. pellucida* is shown in Table 2. The ethyl acetate extract resulted in cell viability (in %) of more than 100% at the tested concentrations [37]. The cells viability was not significantly different from the vehicle control (*p* > 0.05). All of the samples at this concentration were considered non-toxic and were not expected to interfere with the results of the proliferative effect of samples in the E-SCREEN assay.

### 2.3. E-SCREEN Assay

**Figure 3:**
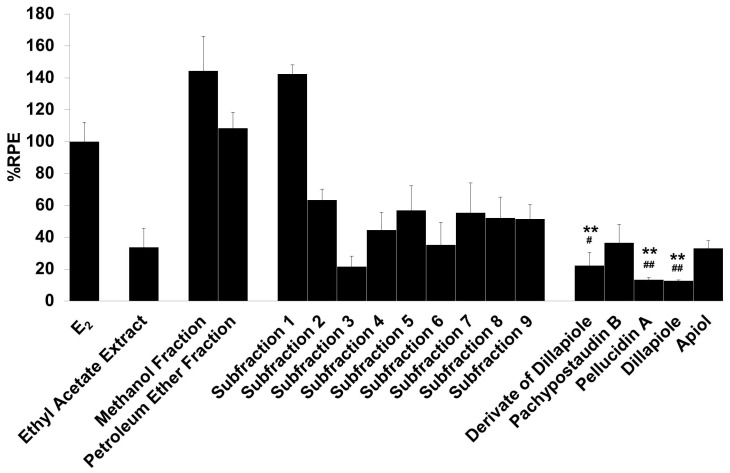
The estrogenic activity of the ethyl acetate extract and its fractions, subfractions, and isolated compounds from *P. pellucida* was measured using the E-SCREEN assay. Cells were treated with 1 µg/mL of the samples for 144 h, and the proliferative effects relative to cells in the presence of E_2_ (10^−9^ M, 100%) were then investigated using sulforhodamine b (SRB) assays. The data are expressed as the mean ± SD of at least two separate experiments with duplicates or triplicates for each group. The symbol ** represents statistically significant differences compared with the methanol fraction, and ## represents statistically significant differences compared with subfraction 1, analyzed by the Kruskal–Wallis test using Bonferroni adjustment; ** *p* < 0.01, # *p* < 0.05, ## *p* < 0.01.

The E-SCREEN assay was used to determine the estrogenic activity of the test samples. The assay assesses the estrogenicity of chemicals based on the proliferative effect of estrogens on their target cells as an endpoint. This quantitative assay compares the cell number for a similar inoculation amount of MCF-7/BUS cells in the absence of estrogens (negative control) and in the presence of 17β-estradiol (positive control) for a range of concentrations of chemicals suspected to be estrogenic. The estrogenic effect was expressed as %RPE, which indicates whether the compound being tested induces a proliferative response that is quantitatively similar to that obtained with E_2_, that is, a full agonist (RPE = 100%) [38]. Figure 3 shows the estrogenic activity of the ethyl acetate extract and its fractions, subfractions, and isolated compounds.

The samples had varying estrogenic activity strength and had significantly higher estrogenic activity than the vehicle control (cells treated with 0.1% DMSO), except for the ethyl acetate extract and subfractions 2, 3, 4, and 6 (*p* < 0.01). Full agonist effects (%RPE > 80%) were observed for the fractions and subfraction 1, while subfraction 3, a derivate of dillapiole, pellucidin A and dillapiole, induced a low estrogenic effect (%RPE < 25%). A full agonist demonstrates a higher capacity to induce MCF-7/BUS cell proliferation [39]. Thus, at the same concentration, the estrogenic effects of the other samples were classified as partial agonist effects (%RPE from 25% up to 80%). The separation processes produced some fractions and subfractions that had higher estrogenic effects than the extract. However, further purification of the methanol fraction did not produce samples with higher activity.

The compounds had lower activity than either their original fractions or subfractions. In comparison with the methanol fraction, a derivate of dillapiole, pellucidin A and dillapiole had significantly lower activity (*p* < 0.01). Subfraction 1 also had superior activity to these compounds (*p* < 0.05 for derivate of dillapiole and *p* < 0.01 for pellucidin A and dillapiole). Polyphenolic compounds detected in the ethyl acetate extract of *P. pellucida* might be responsible for these results because, chemically, phytoestrogens are phenolic phytochemicals or polyphenols [40,41].

**Figure 4:**
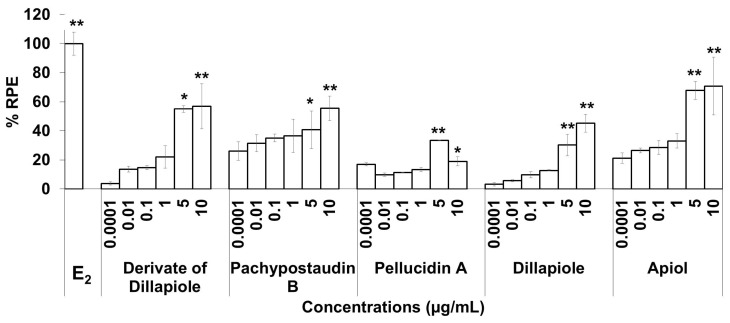
The estrogenic activity of five compounds isolated from the ethyl acetate extract of *P. pellucida* using the E-SCREEN assay. Cells were treated with the compounds in a dose range of 0.001–10 µg/mL for 144 h, and relative proliferative effects on the cells were then investigated using sulforhodamine b (SRB) assays, with 10^−9^ M E_2_ as the standard control. The data are expressed as the mean ± SD of at least two separate experiments with duplicates or triplicates for each group. The symbols * and ** represent statistical differences compared with the vehicle group: * *p* < 0.05, ** *p* < 0.01, analyzed by the Kruskal–Wallis test using Bonferroni adjustment.

The estrogenic activities of the compounds in response to a broad range of doses were compared. The results are shown in Figure 4. The estrogenic activity tended to increase with increasing doses for almost all of the isolated compounds, except pellucidin A. Overall, apiol had higher estrogenic activity than the others, while pellucidin A had the lowest estrogenic activity.

**Figure 5:**
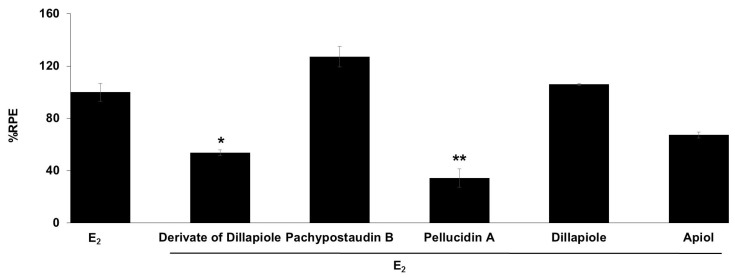
Estrogenic effects of the tested compounds on MCF-7/BUS cells in the presence of 10^−9^ M E_2_. Cells were treated with estradiol alone or estradiol in combination with 10 µg/mL of the tested samples for 144 h, and relative proliferative effects on the cells were then investigated using SRB assays. The data are expressed as the mean ± SD of at least two separate experiments in duplicate or triplicate for each group. The symbols * and ** represent statistical differences compared with the estradiol group: * *p* < 0.05, ** *p* < 0.01, analyzed by the Kruskal–Wallis test using Bonferroni adjustment.

The anti-estrogenic activity of all of the compounds was also determined (Figure 5). Samples are considered to have anti-estrogenicity when they can inhibit cell proliferation induced by 17β-estradiol, compared with the effect of 17β-estradiol alone [39]. The results showed that the derivates of dillapiole and pellucidin A significantly suppressed the estrogenic activity of estradiol, indicating that these compounds may act as ER antagonists or have 17β-estradiol antagonist effects. These results are highly relevant because anti-estrogens block the binding of 17β-estradiol to ERα and antagonize estrogen-stimulated gene expression.

**Figure 6:**
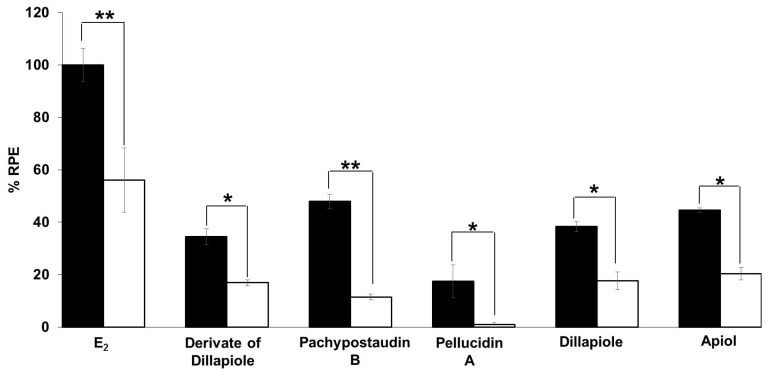
Estrogenic effects of estradiol and the tested compounds in the presence of 10^−6^ M tamoxifen on MCF-7/BUS cells. Cells were treated with estradiol and samples alone or in combination with tamoxifen for 144 h, and then relative proliferative effects on the cells were investigated using SRB assays. The data are expressed as mean ± SD of at least two separate experiments in duplicate or triplicate for each group. Black bars indicate a testing of a single compound in isolation while white bars indicate their combination with tamoxifen. The symbols * and ** represent statistical differences compared with the estrogenic effect of the samples alone: * *p* < 0.05, ** *p* < 0.01, analyzed by the independent-samples t-test or Mann–Whitney U test.

The estrogenic mechanism of the compounds was studied. In this study, the proliferative effects of each estrogenic agent were compared with their effects when used in combination with tamoxifen, an antagonist agent. The results (Figure 6) show that tamoxifen caused significant suppression of the proliferative effect of estradiol and the compounds. In this case, tamoxifen exerted its anti-estrogenic effect. This result shows that the estrogenic activity of the tested samples might be mediated by the same mechanism of action as estradiol, which is related to tamoxifen. This is indicative of a classical ligand-dependent mechanism.

### 2.4. Molecular Docking

Molecular docking was used to identify the interaction of the isolated compounds with ERα and ERβ. In this test, the binding energy and hydrogen bonding profile of isolated compounds with estradiol, a natural ligand, were analyzed and compared.

Molecular docking to ERs revealed that the five compounds demonstrated binding affinity to both receptors (Table 3). However, the binding energies of the compounds were lower than that of estradiol. All compounds had comparable affinities toward both receptors, except for compounds **2** and **3**, which had preferentially bound ERα. The isolated compounds were observed to bind various amino acid residues, especially in ERβ. Overall, compound **2** had the highest binding energy and similarity of amino acid residues compared with other compounds. The molecular docking data were also supported by the visualization of amino acid residues in the binding site (Figures S10 and S11, Supplementary Materials).

## 3. Discussion

In this study, the estrogenic activity of the ethyl acetate extract of *P. pellucida* and its compounds was investigated. This extract was deemed interesting because it produced a partial agonist effect (%RPE of 25%–80%) in our previous study.

SERM drugs are also known to act as partial estrogen receptor agonists for maintaining bone density during osteoporosis treatment while acting as antagonists of the estrogen receptor in breast tissues for breast cancer prevention [42]. These drugs can work selectively and also play a role as antagonists when a full agonist is present. This agent may either block or stimulate the expression of certain genes [43,44]. Besides acting as a partial agonist, phytoestrogens also can act as an agonist with low estrogenic activity or an antagonist for ERs, inducing the expression of estrogen-responsive gene products. When acting on estrogen receptors, they behave differently from estrogen and act like SERMs [45,46,47,48].

An extract usually contains numerous compounds that might interact with each other to produce an observed effect. In this study, the ethyl acetate extract was separated and purified under the guidance of the E-SCREEN bioassay to isolate several compounds with potential estrogenic activity. Then, the estrogenic activities of the ethyl acetate extract and its methanol and petroleum ether fractions, subfractions (1–9), and five isolated compounds were compared.

In this study, compound **1**, a new derivate of dillapiole (6-allyl-5-methoxy-1,3-benzodioxol-4-ol), and **2**, pachypostaudin B, were isolated from *P. pellucida* for the first time. Previously, pachypostaudin B was isolated from the bark of *Pachypodanthium staudlii* Engl. and Diels and *Pachypodanthium confine* Engl. and Diels [35,49]. The compound is considered a chemotaxonomic marker for the Annonaceae family, given its unique occurrence [32]. No activity related to these compounds has been reported. Although compound **2** has been reported as an antiviral agent against polio, there are no adequate data to support this assertion [29,35].

The isolation of the other compounds (**3**–**5**) has been reported in previous studies. Pellucidin A and dillapiole were isolated from the chloroform extract of *P. pellucida* and apiol was isolated from its ether-soluble neutral fraction [34,50]. Pellucidin A from *P. pellucida* was reported to have antimutagenic and antifungal effects against *Trichophyton mentagrophytes* [34]. Dillapiole has numerous reported effects; it has been found to act as a cytochrome P450 3A4 inhibitor, antibacterial agent, insecticide, antifungal agent, and anti-inflammatory agent in addition to DNA adduction activity [51,52,53,54,55,56,57]. Dillapiole that was isolated from *P. pellucida* was reported to have a gastroprotective effect [58]. Apiol was demonstrated to have an acaricidal effect [59], DNA adduction effect [57], and anti-proliferative effect [60]. The anti-proliferative effect of apiol isolated from *Athamanta sicula* L. was reported against K-562 (human chronic myelogenous leukemia), NCI-H460 (human lung tumor), and MCF-7 cell lines. Apiol was reported to have 100% inhibition toward the MCF-7 cell line at a concentration of 100 µg/mL with an IC_50_ of 36 µg/mL in these studies [57,60]. Thus, this study is the first report of the estrogenic activity of these isolated compounds (**1**–**5**).

The methanol fraction had the highest estrogenic activity among the tested samples. The higher estrogenic activity of the methanol fraction relative to the petroleum ether fraction indicates that the estrogenic activity is mostly due to polar–semipolar compounds. This finding is in accordance with a previous study [61]. Even an anti-estrogenic agent like tamoxifen must undergo bioactivation to become hydroxytamoxifen, which is more polar, to have a high affinity for the estrogen receptor [62].

All of the compounds had lower estrogenic activity than their original fractions and subfractions. This finding indicates that the estrogenic activity of fractions and subfractions, or even the extract, might be produced by interactions between the compounds therein.

As mentioned in the results section, all of the isolated compounds are classified as lignans. In addition to isoflavones, lignans are one of the primary groups of phytoestrogen compounds. Lignans, including enterolactone and enterodiol, present weak estrogenic activity [46,63]. A previous study reported the successful isolation of one lignan compound from *P. pellucida* that has estrogenic activity [64]. Lignan-type compounds might greatly contribute to the estrogenic activity observed for the ethyl acetate extract of *P. pellucida*.

The compounds isolated in this study exhibited different estrogenic activity profiles. Apiol had the highest estrogenic activity, while pellucidin A had the lowest. The estrogenic activity tended to increase with increasing doses, except for pellucidin A, whose activity began to decrease at a dose of 10 µg/mL. Only the derivates of dillapiole and pellucidin A exerted significant anti-estrogenic effects. The presence of both estrogenic and anti-estrogenic activity was supported by the molecular docking data. The data show that all of the compounds have a binding affinity to both ERs.

ERs are present in various organs in the body. ERα and ERβ activities have been found to be antagonistic toward each other in many tissues. The activation of ERα promotes cell proliferation and survival, whereas ERβ produces protective or anti-proliferative and pro-apoptotic effects in the prostate and breast [3,4,65]. In a similar manner, an anti-proliferative effect was also found to be exerted by ERβ in the brain, colon, and lung [66,67,68,69,70,71]. In bone, estrogen can induce osteoclast apoptosis only via ERα [72,73].

The estrogenic activity of the test compounds might be mediated by the same mechanism as estradiol because it decreased with estradiol when combined with tamoxifen. Tamoxifen acts as an anti-estrogen agent in the breast due to its competitive inhibition against estrogen, the recruitment of co-repressors, and inhibition of the constitutive activation function-2 (AF-2). AF-2 is a nonacidic activation domain that is located in the ligand-binding domain of estrogen and has a role in mediating the ligand-dependent mechanism of estrogen [74,75,76]. Tamoxifen is known to prevent breast cancer by stimulating estrogen through both genomic and non-genomic mechanisms [77,78]. Hence, the mechanism of action of the samples might be related to those mechanisms, especially the classical ligand-dependent mechanism.

Overall, the partial agonist effect of the ethyl acetate extract might be produced by the interaction of the compounds. The compounds might work individually, synergistically, or antagonistically or as described in other studies [79,80]. To confirm that the mechanism of action of the compounds occurs via the classical ligand-dependent mechanism, the binding capability of compounds to ERs was characterized using molecular docking. Some compounds had a binding affinity toward the same or different amino acid residues. For example, **3** and **5** have different types of amino acid residues that bind to Erα, suggesting the presence of individual or synergistic action. By contrast, **2** and **3** have similar binding energies, amino acid residues for binding, and H-bond distance, suggesting the possibility that they antagonize each other or even estrogen. This possibility needs to be investigated further.

The high anti-estrogenic activity of pellucidin A might also be mediated by mechanisms other than the classical ligand-dependent mechanism. This was considered in light of its low binding affinity to ERβ. This compound might be able to block the nuclear uptake of the receptor and then inhibit its nucleocytoplasmic shuttling. In this case, the anti-estrogenic agent can bind to ER to form complexes and then bind to estrogen response elements (EREs), leading to the inactivation of the transcriptional unit [81]. This effect is stronger for higher doses of the compound, as shown in Figure 4.

In this study, the compounds isolated from *P. pellucida* were found to have different estrogenic profiles. Their effects on osteoporosis should be assessed in a future study to determine whether they also show good anti-osteoporosis activity as might be expected from an agent with estrogenic activity. Estrogenic agents are known to have effects on osteoclasts, osteocytes, and osteoblasts, leading to decreased bone resorption, inhibition of bone remodeling, and maintenance of bone formation, respectively [15]. The safety of the compounds should also be examined because the estrogenic activity can detrimentally affect not only breast organs and bone but also other estrogen-responsive organs.

## 4. Materials and Methods

### 4.1. Instruments and Materials

Column chromatography was performed on silica (Si) gel 60 (230–400 mesh, Merck, Darmstadt, Germany), LiChroprep RP-18 (40–63 μm, Merck, Kenilworth, NJ, USA), Sephadex LH-20 (25–100 μm, Sigma-Aldrich, St. Louis, MO, USA), and a Lobar column, while thin-layer chromatography (TLC) was carried out on precoated silica gel 60 F_254_ plates (Merck) and RP-18 F_254S_ plates (Merck). Nuclear magnetic resonance (NMR) experiments were performed on Bruker AVANCE III HD 850, AVANCE III 600 and Ascend^TM^ 400 spectrometers with the usual pulse sequences.

*P. pellucida* plants (wild) were collected from Cagak and Ciater Region, West Java Province, Indonesia, in March–April 2016. The plant was authenticated by a botanist at the Herbarium Bandungense, Bandung Institute of Technology, Indonesia, with document number 705/I1.CO2.2/PL/2016. As extraction solvents, n-hexane and ethyl acetate were provided by CV Fadillah (Bandung Kulon, Indonesia). Fetal bovine serum (FBS) was purchased from HyClone (Logan, UT, USA). Dulbecco’s modified Eagle’s Medium (DMEM) and trypsin-EDTA were purchased from Invitrogen (Carlsbad, CA, USA). WST-1 reagent was purchased from Roche, Mannheim, Germany. Penicillin and streptomycin were obtained from GibcoBRL (Grand Island, NY, USA). Dimethyl sulfoxide (DMSO), quercetin, stigmasterol, Na_2_CO_3_, 17β-estradiol, and tamoxifen were purchased from Sigma-Aldrich (St. Louis, MO, USA).

### 4.2. Extraction Process

The ethyl acetate extract that was used in this study was prepared using a sequential maceration technique for 3 × 24 h following the extraction of 500 g of the dry plant by n-hexane. The residue of the n-hexane extract was dried and extracted with 10 L of ethyl acetate. The filtrate was collected and evaporated using a rotary evaporator.

### 4.3. Isolation Process

In this study, we successfully isolated 5 compounds from the ethyl acetate extract of *P. pellucida*. Two of them were newly isolated compounds from this plant. We used several methods to isolate these compounds. The compounds were named according to the order of completion of the isolation process.

The isolation process started with the fractionation of ethyl acetate extract (6 g) using 70% methanol and petroleum ether through a liquid–liquid chromatography method. Two fractions were obtained from this process. The methanol fraction (1.475 g) was further fractionated using a Si gel column with 100% n-hexane, n-hexane, and ethyl acetate (7:3), n-hexane and ethyl acetate (1:1), n-hexane and ethyl acetate (3:7), 100% ethyl acetate, ethyl acetate and ethanol (1:1), and 100% ethanol as mobile phases, consecutively. Based on the TLC profiles, the subfractions were either separated or combined into 9 subfractions: F1 (31.3 mg), F2 (97.6 mg), F3 (55.2 mg), F4 (67.3 mg), F5 (72.9 mg), F6 (37.9 mg), F7 (72.6 mg), F8 (40.5 mg), and F9 (471.1 mg). We decided to combine F1 and F2 according to activity test results and their similar TLC profiles. This combined fraction was separated further using an RP-C18 column with 70%–100% methanol as mobile phases. From this process, 20 subfractions were obtained. According to the TLC profiles, we combined several fractions with unique characteristics and tried to isolate potential compounds.

The combined subfractions F6 and F7, with a total weight of 10.5 mg, were fractionated using a Lobar column with n-hexane, methylene chloride, and methanol in a ratio of 10:10:0.3 as the mobile phase. The 3rd subfraction was separated further using a Sephadex LH-20 column with methanol as the mobile phase. The 3rd subfraction from that process was used to isolate compound **1** (2.4 mg).

F10 was separated using a Sephadex LH-20 column with a mobile phase consisting of methylene chloride and methanol in an equal ratio. Compound **2** (0.8 mg) was obtained through crystallization of the 3rd subfraction using methanol as a solvent.

The combined subfractions F11 (5.6 mg) and F12 (4.0 mg) were separated using a Sephadex LH-20 column with methanol as the mobile phase. The 4th fraction from this process was used to isolate compound **3** (3.4 mg) through crystallization with methanol as a solvent.

Compounds **4** and **5** were isolated from the ethyl acetate extract (8.67 g). The ethyl acetate extract was fractionated using a Si gel column with several combinations of mobile phases: 100% n-hexane; n-hexane and ethyl acetate (10:1); n-hexane and ethyl acetate (1:1); n-hexane, ethyl acetate, and methanol (10:10:1); n-hexane, ethyl acetate, methanol (10:10:5); ethyl acetate, methanol, water (10:5:3); ethyl acetate, methanol, water (10:10:7); methylene chloride and methanol (3:1); methylene chloride and methanol (1:1); and then 100% methanol, consecutively.

Based on the TLC profile, the fractions were either separated or combined, resulting in 12 fractions: F1 (68.9 mg), F2 (9 mg), F3 (3.6 mg), F4 (522.5 mg), F5 (954.6 mg), F6 (493.1 mg), F8 (771.7 mg), F9 (751.2 mg), F10 (1.016 g), F11 (2.051 g), and F12 (274.3 mg). Fraction 4 was chosen for further fractionation. This fraction was separated using column chromatography on RP-C18. Further purification of the second subfraction from this separation process was performed on a recycling preparative HPLC instrument to obtain compounds **4** (61.2 mg) and **5** (4.4 mg). The complete isolation procedures were shown in Figure S1 Supplementary Materials.

Data from ^1^H and ^13^C-NMR were used to support the identification of the isolated compounds (Figures S2–S9, Supplementary Materials). ^1^H and ^13^C NMR spectra were recorded at 400 and 213.8/100 MHz in CDCl_3_, respectively except for compound **2** spectra which were recorded at 600 and 150.9 MHz. Compound **2** was considered as an interesting compound, thus it was analyzed further using 2D NMR including Distortionless Enhancement by Polarization Transfer (DEPT), Correlation spectroscopy (COSY), Nuclear Overhauser Effect Spectroscopy (NOESY) and Heteronuclear Multiple Bond Correlation Spectroscopy (HMBC). ChemSpider^®^ was used to find information about the compounds.

### 4.4. Cell Lines

The estrogen-sensitive MCF-7/BUS human breast cancer cells used in the E-SCREEN assay were kindly provided by Dr. Soto (Tufts University, Boston, MA, USA). Phenol Red DMEM supplemented with 5% fetal bovine serum, penicillin (100 units/mL), and streptomycin (100 µg/mL) was used as the growth medium. The cells were incubated in a humidified incubator at 37 °C and 5% CO_2_/95% air.

### 4.5. Cytotoxicity Assay

Cytotoxicity was evaluated using the WST-1 cell proliferation assay as previously described [82]. The concentration of samples, especially extracts and fractions, as µg/mL were determined by considering the whole content of the samples. First, a stock solution containing 10,000 µg/mL of each sample was prepared by suspending a particular amount of samples in an appropriate volume of acetone as a vehicle, such as 2 mg sample in 200 µL acetone, followed by vortex mixing. From the stock solution, serially diluted solutions of concentrations 10 µg/mL, 100 µg/mL, and 1000 µg /mL were prepared (solution A). Then, distilled water was used to make another serially diluted solutions of concentrations 1 µg/mL, 10 µg/mL, and 100 µg/mL from solution A (solution B with 10% acetone). Finally, serial diluted solutions of concentrations 0.1 µg/mL, 1 µg /mL, and 10 µg mL were prepared by mixing solution B and medium to provide assay solutions, which contained 1% acetone. The acetone concentration used in this study was considered non-toxic to the cells based on our preliminary study supported by other literature [83,84]. At the end of the additional incubation period, all medium was removed and centrifuged at 8000 rpm for 4 min at room temperature. The absorbance of 80 µL of the supernatant was measured by using a VERSAmax microplate reader (Molecular Devices, Sunnyvale, CA, USA) at 440 nm versus a 690 nm reference. Cell viability (in %) was calculated using the following formula: (%) = [(sample absorbance)/(absorbance of vehicle control) × 100].

### 4.6. E-SCREEN Assay

The E-SCREEN assay was conducted as previously described [85]. To determine the estrogenic activity, the samples (extract, fractions, subfractions, and isolated compounds) were added to the experimental medium at certain concentrations of the medium. Estradiol (10^−9^ M, E_2_) was added to 1 µg/mL of samples in order to determine anti-estrogenic activity. Furthermore, in order to determine the mechanism of action of the samples, the samples were combined with 10^−6^ M tamoxifen. Absorbances were measured using a VERSAmax microplate reader (Molecular Devices, Sunnyvale, CA, USA). The level of estrogenic activity is expressed as %RPE (relative proliferative effect), which was calculated as follows: %RPE = [(S − 1)/(E − 1)] × 100, where S = proliferation of samples and E = proliferation of positive control (10^−10^ M E_2_). Excel (Microsoft, New York, NY, USA) was used for the calculation of these parameters based on the indicated formulae and functions.

### 4.7. Molecular Docking Test

The target for the estrogenic activity of the compounds was the orientation of ligand bound to the active sites of estrogen receptor alpha (ERα) and beta (ERβ), which were determined using automated docking. The protein structure files were taken from PDB (www.rcsb.org/pdb) (PDB ID: 1GWR for ERα with a resolution of 2.4 Å and 3OLS for ERβ with a resolution 2.2 Å). Discovery Studio was used to edit the proteins by removing heteroatoms and water molecules. The study used Avogadro to geometrically optimize the ligands. Autodock Tools 4.0 was employed, and the Lamarckian genetic algorithm method was implemented. Autodock4 and Autogrid4 were used to calculate docking parameters. The grid map was centered at particular residues of the protein, which were generated with Autogrid. All torsions were allowed to rotate during the docking process.

### 4.8. Data Analysis

SPSS software version 22.0 for Windows was used to perform statistical analyses. Statistical analyses were performed by one-way analysis of variance (ANOVA) or Student’s t-test for normally distributed data. Non-normally distributed data were analyzed using the Kruskal–Wallis test with Bonferroni adjustment or the Mann–Whitney U test. The values for the samples were considered significant at *p* < 0.05 or *p* < 0.01. The data from each assay were expressed as the mean ± standard deviation (SD).

## 5. Conclusions

In conclusion, the ethyl acetate extract from *P. pellucida* might produce a partial agonist effect through the interaction of its constituent compounds. The isolated compounds were determined to exert their estrogenic effects through their binding affinity to ERs in a classical ligand-dependent mechanism. Among the isolated compounds, compound **2**, pachypostaudin B, which was first isolated from *P. pellucida*, seems to have promising potency as an estrogenic agent, especially for osteoporosis treatment. Future studies are also warranted to better define the efficacy and safety of the isolated compounds on target tissue where ERs are present.

## Figures and Tables

**Table 1:** ^1^H (600 MHz), ^13^C (150.9 MHz), DEPT, COSY, NOESY, and HMBC NMR data (CDCl_3_) of pachypostaudin B (**2**).

Carbon Position	DEPT	HSQC		COSY	NOESY	HMBC
	C/H	^13^C	^1^H			
1	CH	28.5	4.89 (1H, *d,* 8.11 Hz)	H-2		C-2, 3, 8, 9, 10, 1a, 2a, 6a
2	CH_2_	29.9	2.42/2.66 (2H, *m*)		H-1	C-3, 4, 1a
3	CH	123.7	5.68 (1H, *m*)	H-2	H-4	C-2, 10
4	CH	121.3	6.82 (1H, *dd,* 3.19, 9.75 Hz)	H-2, 3		C-2
5	C	151.7				
6	CH	96.0	6.44 (1H, *s*)	H-OMe		C-5, 7, 8, 10
7	C	152.6				
8	C	140.4				
9	C	124.6				
10	C	117.1				
1a	C	132.4				
2a	C	150.8				
3a	CH	98.0	6.30 (1H, *s*)	H-OMe		C-2a, 4a
4a	C	148.0				
5a	C	142.5				
6a	CH	114.4	6.53 (1H, *s*)			C-1, 2a, 4a, 5a
8-OMe	CH_3_	60.6	3.39 (3H, s)			C-8
5a-OMe	CH_3_	57.0	3.57 (3H, s)		H-6a	C-5a
4xOMe	CH_3_	56.9, 56.4, 56.2, 56.1	3.84, 3.86, 3.87, 3.91 (each 3H, *s*)		H-6, 3a	C-5, 7, 2a, 4a

**Table 2:** Viability test results of tested samples from *P. pellucida* using the WST-1 cell proliferation assay.

Group of Samples	Cell Viability (in%, Mean ± SD)								
	0.1 µg/mL			1 µg/mL			10 µg/mL		
Methanol Fraction	97.15	±	14.97	114.01	±	48.88	118.99	±	30.94
Petroleum Ether Fraction	67.67	±	8.09	72.33	±	8.78	76.60	±	12.25
Subfraction 1	101.75	±	15.31	104.48	±	18.09	106.23	±	17.42
Subfraction 2	103.15	±	52.06	104.41	±	12.63	108.90	±	23.77
Subfraction 3	118.42	±	6.28	97.23	±	10.37	81.20	±	9.80
Subfraction 4	118.66	±	19.22	95.83	±	12.79	87.17	±	0.17
Subfraction 5	80.47	±	21.69	91.55	±	20.19	97.91	±	29.11
Subfraction 6	124.44	±	23.11	106.25	±	0.67	101.85	±	47.25
Subfraction 7	104.56	±	12.10	107.05	±	30.85	115.53	±	18.68
Subfraction 8	108.36	±	0.87	109.53	±	17.14	112.90	±	33.80
Subfraction 9	111.34	±	6.68	110.83	±	6.07	98.07	±	27.05
Derivate of Dillapiole	113.95	±	18.36	107.08	±	19.05	101.65	±	19.26
Pachypostaudin B	105.43	±	12.92	86.94	±	8.93	82.06	±	14.77
Pellucidin A	88.82	±	2.65	107.92	±	11.05	125.56	±	23.10
Dillapiole	89.88	±	19.04	105.00	±	11.97	104.96	±	4.49
Apiol	116.88	±	5.39	110.04	±	33.57	104.68	±	23.58

**Table 3:** Molecular docking data for binding of estradiol and five isolated compounds of *P. pellucida* to estrogen receptors.

Compounds	Hydrogen Bonding (ER α)			Hydrogen Bonding (ER β)		
	ΔG Bind (kcal/mol)	Amino Acid Residues	H-Bond Distance (Å)	ΔG Bind (kcal/mol)	Amino Acid Residues	H-Bond Distance (Å)
17β-Estradiol	−9.55	ARG 394	2.26	−9.55	ARG 346	2.05
		HIS 524	2.24		HIS 475	2.16
Derivate of Dillapiole	−5.45	-	-	−5.29	LEU 298	2.09
Pachypostaudin B	−8.17	HIS-524	3.02	−7.03	HIS-475	1.91
					LEU-476	2.74
Pellucidin A	−8.20	HIS-524	3.15	−7.65	-	-
Dillapiole	−5.14	-	-	−5.26	LEU 476	3.09
Apiol	−5.58	ARG-394	3.01	−5.44	ARG 346	2.11

## Supplementary Materials

The following are available online. The NMR data of compounds are available as supporting information.
