# Supplementary material for: Isolation and Characterization of Phenylpropanoid and Lignan Compounds from Peperomia pellucida [L.] Kunth with Estrogenic Activities
Source: Molecules. 2020 Oct 23;25(21):4914. doi: 10.3390/molecules25214914 (PMC7660628; doi:10.3390/molecules25214914)
Supplement: Supplementary file 1 [file molecules-25-04914-s001.pdf]

Supporting Information

for

Isolation and Characterization of Phenylpropanoid and Lignan Compounds from *Peperomia pellucida* [L.] Kunth with Estrogenic Activities

Kartika I Gusti Agung Ayu, In Jae Bang, Riani Catur , Insanu Muhamad, Jong Hwan Kwak*, Kyu Hyuck Chung*, Adnyana I Ketut*

School of Pharmacy, Institut Teknologi Bandung, Bandung 40132, Indonesia

School of Pharmacy, Sungkyunkwan University, Suwon 16419, Republic of Korea

Corresponding authors:

*(J.H.K.) Tel: +82-31-290-7745. Fax: +82-31-292-8800. E-mail: jhkwak@skku.edu.

*(K.H.C.) Tel: +82-31-290-7714. Fax: +82-31-290-7771. E-mail: khchung@skku.edu.

*(I.K.A.) Telp: +62-822-1672-3162. Fax: +62-22 2504852. Email: ketut@fa.itb.ac.id

List of Contents

Figure S1. Isolation process of compound 1-5.

Figure S2. ^{1}H and ^{13}C NMR spectra of compound **1** ($CDCl_3$, 400 and 213.8 MHz).

Figure S3. ^{1}H and ^{13}C NMR spectra of compound **2** ($CDCl_3$, 600 and 150.9 MHz).

Figure S4. DEPT spectra of compound **2**

Figure S5. ^{1}H-^{1}H COSY and NOESY spectra of compound **2**

Figure S6. HSQC and HMBC spectra of compound **2**

Figure S7. ^{1}H and ^{13}C NMR spectra of compound **3** ($CDCl_3$, 400 and 213.8 MHz)

Figure S8. ^{1}H and ^{13}C NMR spectra of compound **4** ($CDCl_3$, 400 and 100 MHz)

Figure S9. ^{1}H and ^{13}C NMR spectra of compound **5** ($CDCl_3$, 400 and 213.8 MHz)

Figure S10. Ligand-Receptor Interaction of Compound **1-5** and Estrogen Receptor α

Figure S11. Ligand-Receptor Interaction of Compound **1-5** and Estrogen Receptor β

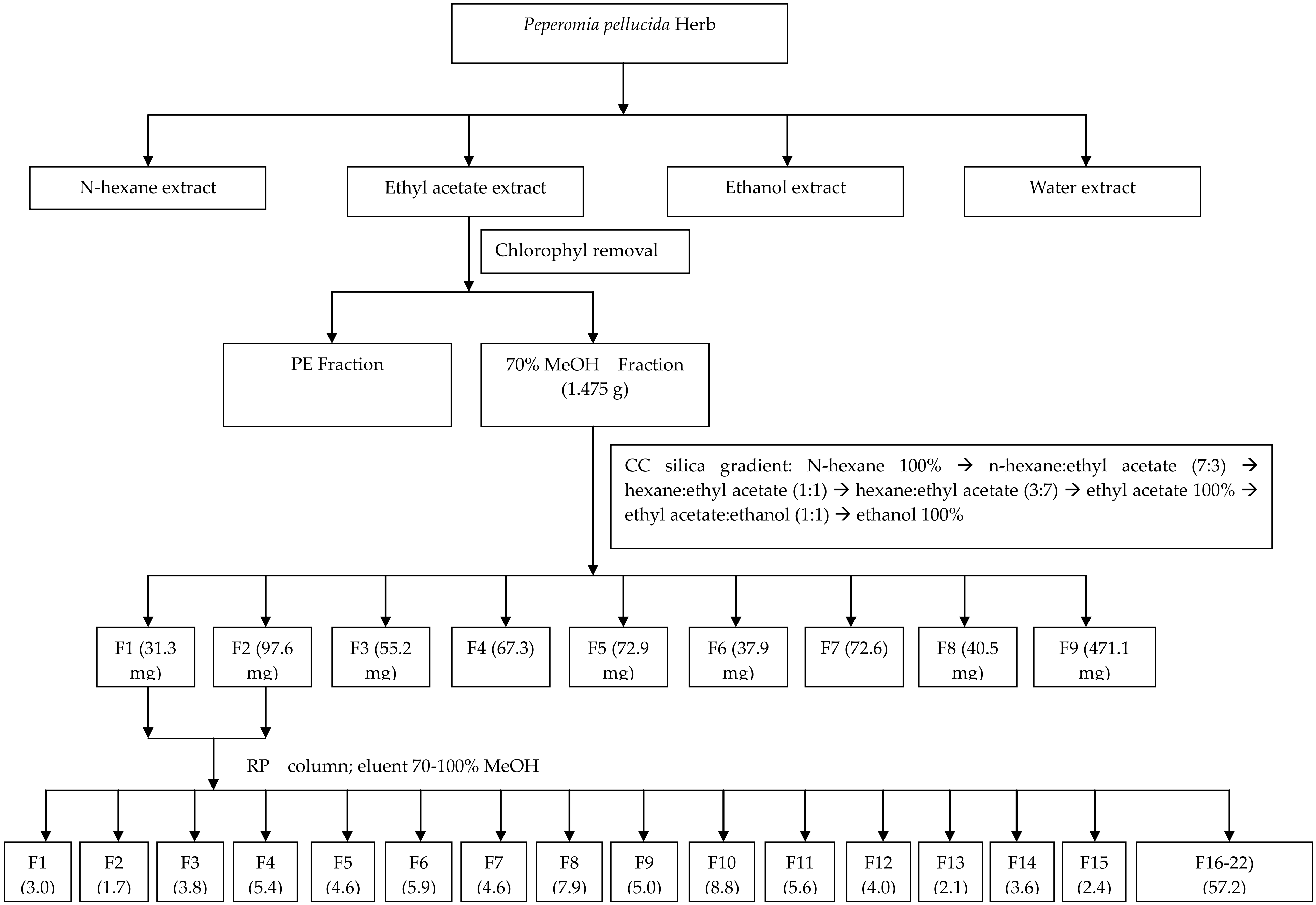

Peperomia pellucida Herb
N-hexane extract
Ethyl acetate extract
Ethanol extract
Water extract
Chlorophyl removal
PE Fraction
70% MeOH Fraction (1.475 g)
CC silica gradient: N-hexane 100% → n-hexane:ethyl acetate (7:3) → hexane:ethyl acetate (1:1) → hexane:ethyl acetate (3:7) → ethyl acetate 100% → ethyl acetate:ethanol (1:1) → ethanol 100%
F1 (31.3 mg)
F2 (97.6 mg)
F3 (55.2 mg)
F4 (67.3)
F5 (72.9 mg)
F6 (37.9 mg)
F7 (72.6)
F8 (40.5 mg)
F9 (471.1 mg)
RP column; eluent 70-100% MeOH
F1 (3.0)
F2 (1.7)
F3 (3.8)
F4 (5.4)
F5 (4.6)
F6 (5.9)
F7 (4.6)
F8 (7.9)
F9 (5.0)
F10 (8.8)
F11 (5.6)
F12 (4.0)
F13 (2.1)
F14 (3.6)
F15 (2.4)
F16-22) (57.2)

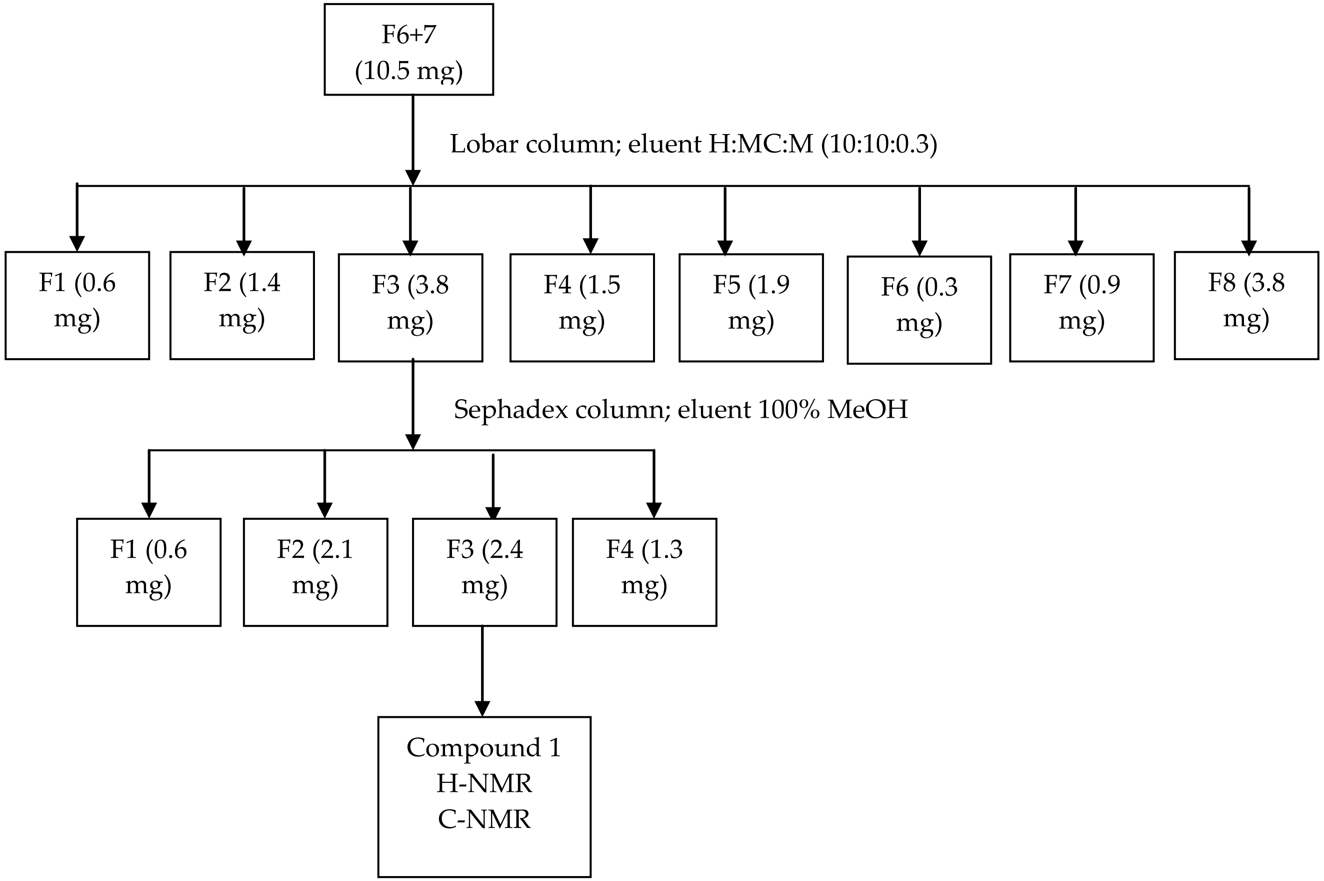
F6+7
(10.5 mg)
Lobar column; eluent H:MC:M (10:10:0.3)
F1 (0.6 mg)
F2 (1.4 mg)
F3 (3.8 mg)
F4 (1.5 mg)
F5 (1.9 mg)
F6 (0.3 mg)
F7 (0.9 mg)
F8 (3.8 mg)
Sephadex column; eluent 100% MeOH
F1 (0.6 mg)
F2 (2.1 mg)
F3 (2.4 mg)
F4 (1.3 mg)
Compound 1
H-NMR
C-NMR

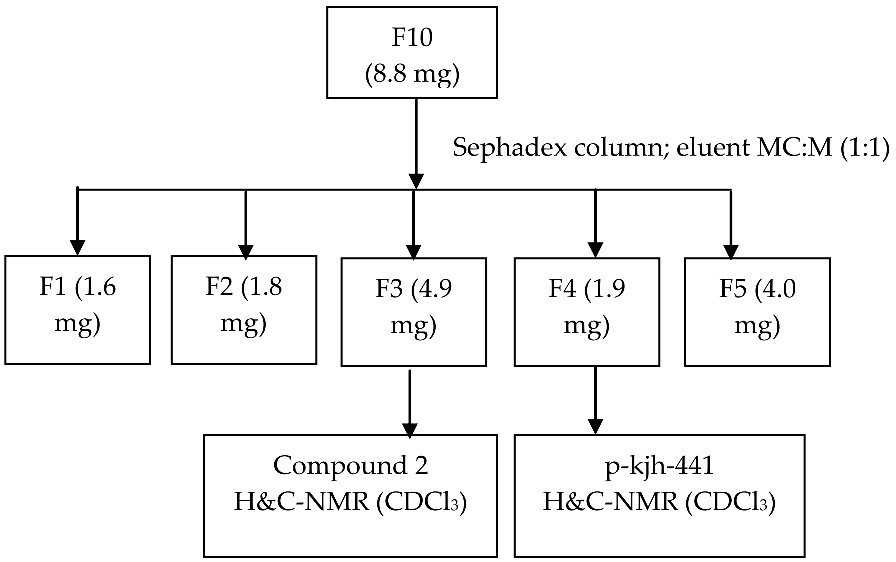
F10
(8.8 mg)
Sephadex column; eluent MC:M (1:1)
F1 (1.6 mg)
F2 (1.8 mg)
F3 (4.9 mg)
F4 (1.9 mg)
F5 (4.0 mg)
Compound 2
H&C-NMR (CDCl3)
p-kjh-441
H&C-NMR (CDCl3)

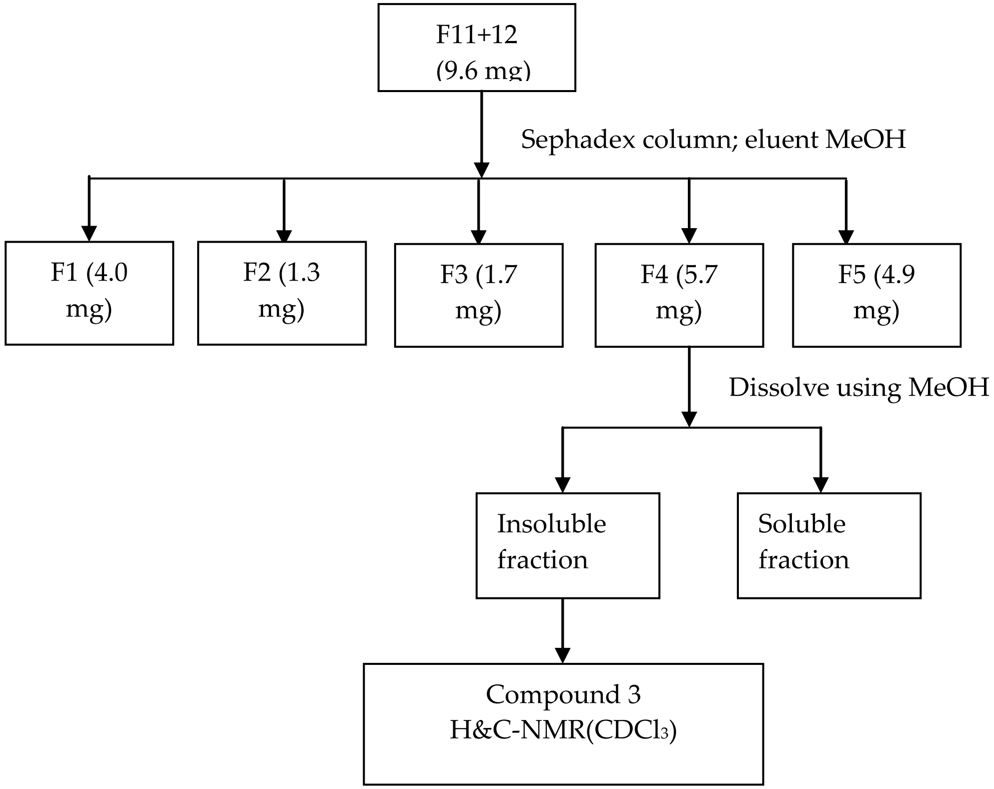
F11+12
(9.6 mg)
Sephadex column; eluent MeOH
F1 (4.0 mg)
F2 (1.3 mg)
F3 (1.7 mg)
F4 (5.7 mg)
F5 (4.9 mg)
Dissolve using MeOH
Insoluble fraction
Soluble fraction
Compound 3
H&C-NMR(CDCl3)

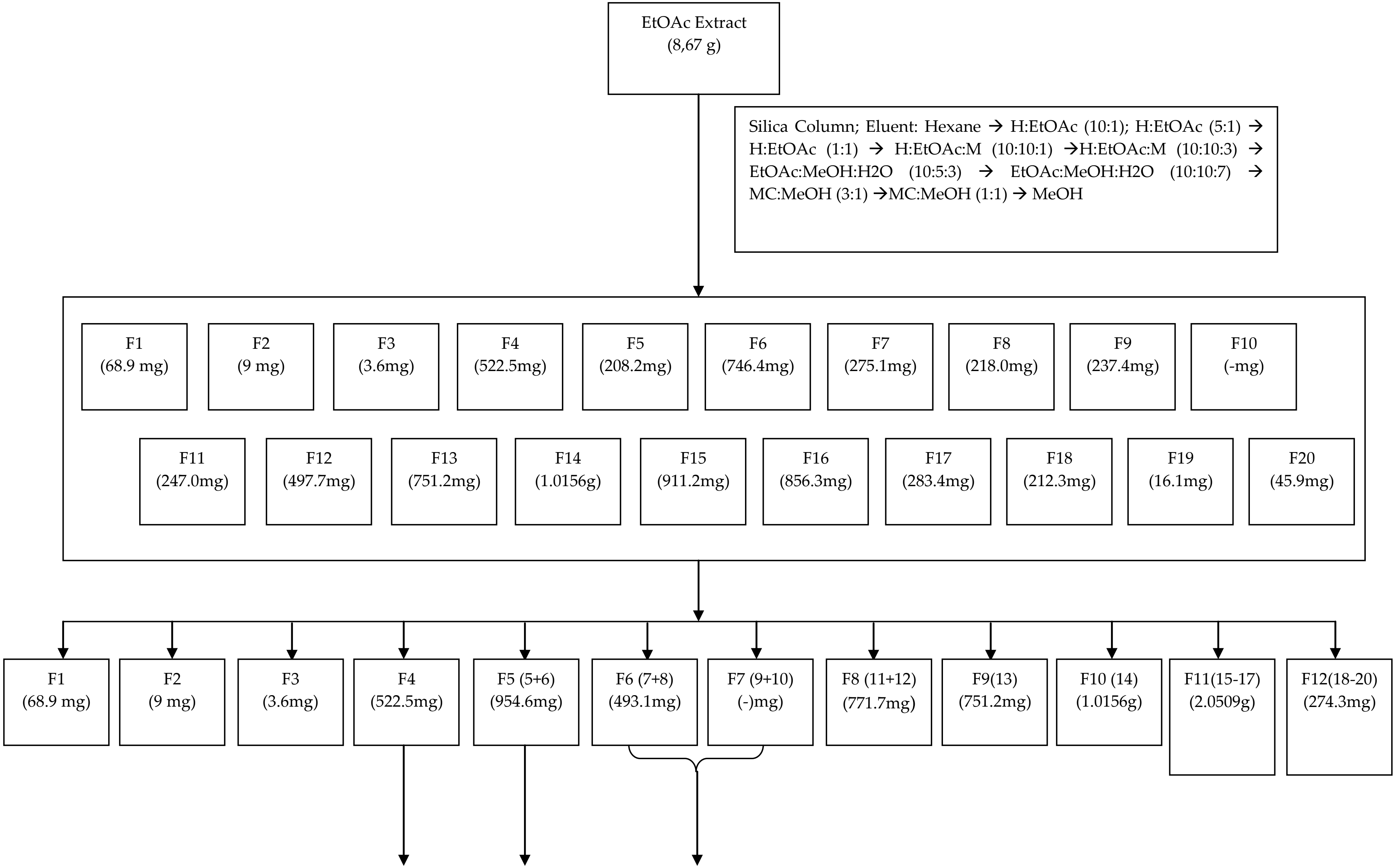
EtOAc Extract
(8,67 g)
Silica Column; Eluent: Hexane → H:EtOAc (10:1); H:EtOAc (5:1) → H:EtOAc (1:1) → H:EtOAc:M (10:10:1) →H:EtOAc:M (10:10:3) → EtOAc:MeOH:H2O (10:5:3) → EtOAc:MeOH:H2O (10:10:7) → MC:MeOH (3:1) →MC:MeOH (1:1) → MeOH
F1 (68.9 mg)
F2 (9 mg)
F3 (3.6mg)
F4 (522.5mg)
F5 (208.2mg)
F6 (746.4mg)
F7 (275.1mg)
F8 (218.0mg)
F9 (237.4mg)
F10 (-mg)
F11 (247.0mg)
F12 (497.7mg)
F13 (751.2mg)
F14 (1.0156g)
F15 (911.2mg)
F16 (856.3mg)
F17 (283.4mg)
F18 (212.3mg)
F19 (16.1mg)
F20 (45.9mg)
F1 (68.9 mg)
F2 (9 mg)
F3 (3.6mg)
F4 (522.5mg)
F5 (5+6) (954.6mg)
F6 (7+8) (493.1mg)
F7 (9+10) (-)mg)
F8 (11+12) (771.7mg)
F9(13) (751.2mg)
F10 (14) (1.0156g)
F11(15-17) (2.0509g)
F12(18-20) (274.3mg)

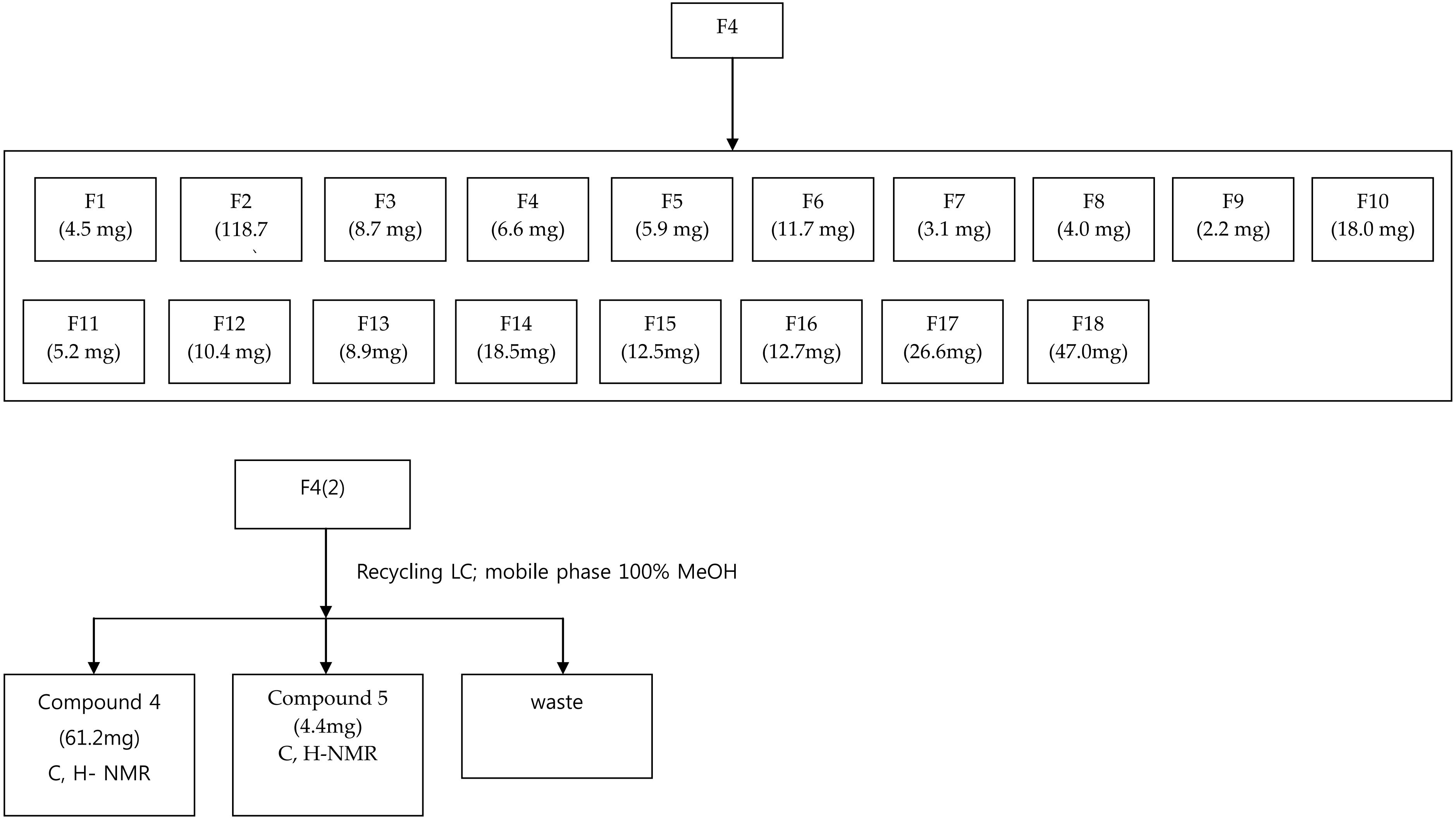

Figure S1. Isolation process of compound 1-5.

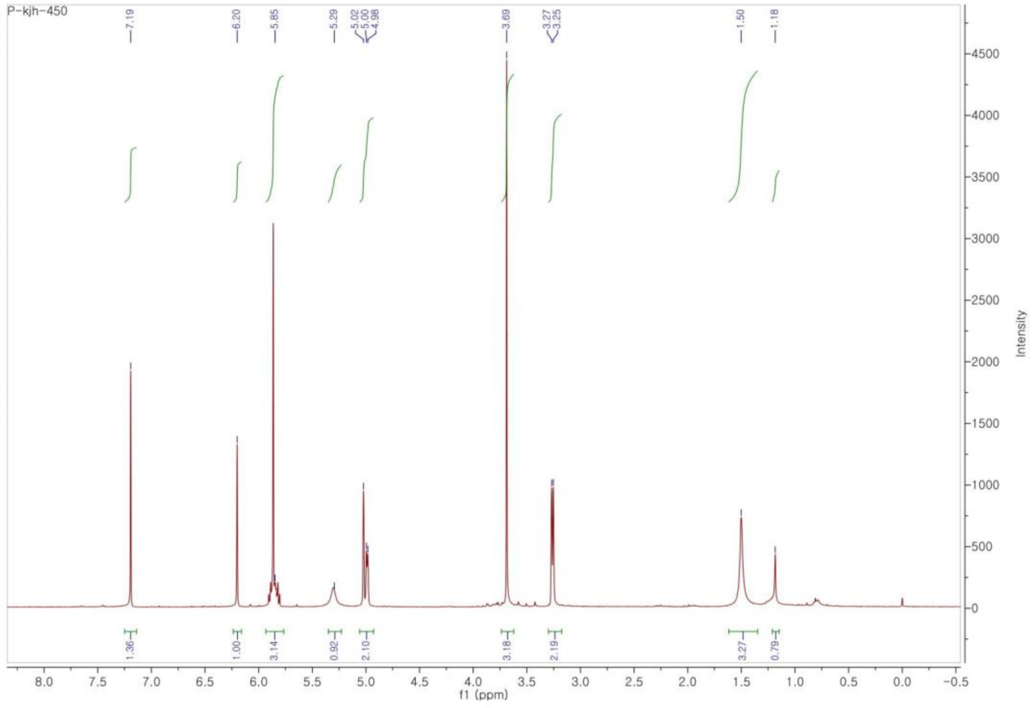

(A)

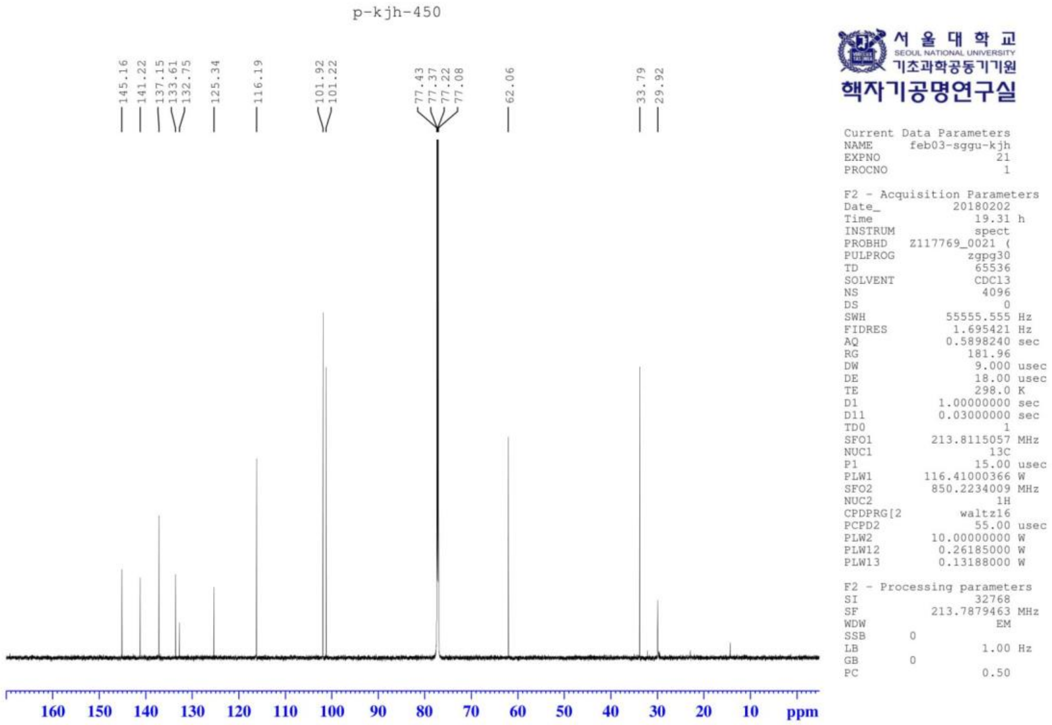

(B)

Figure S2. ^{1}H and ^{13}C NMR spectra of compound **1** ($CDCl_3$, 400 and 213.8 MHz).

(A) ^{1}H NMR spectrum, (B) ^{13}C NMR spectrum

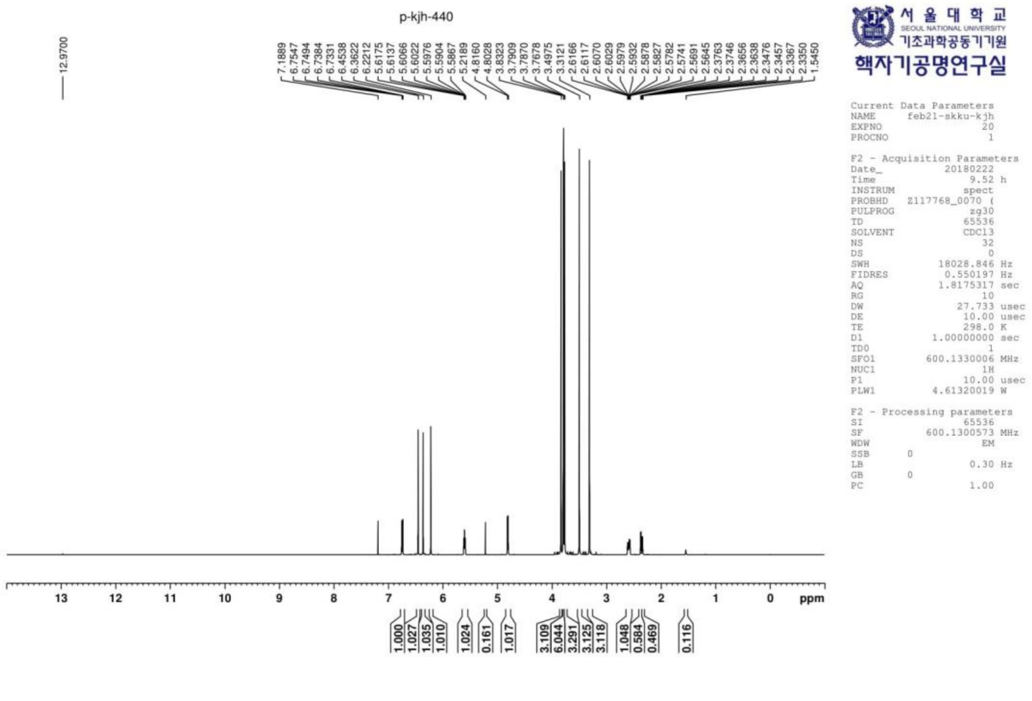

(A)

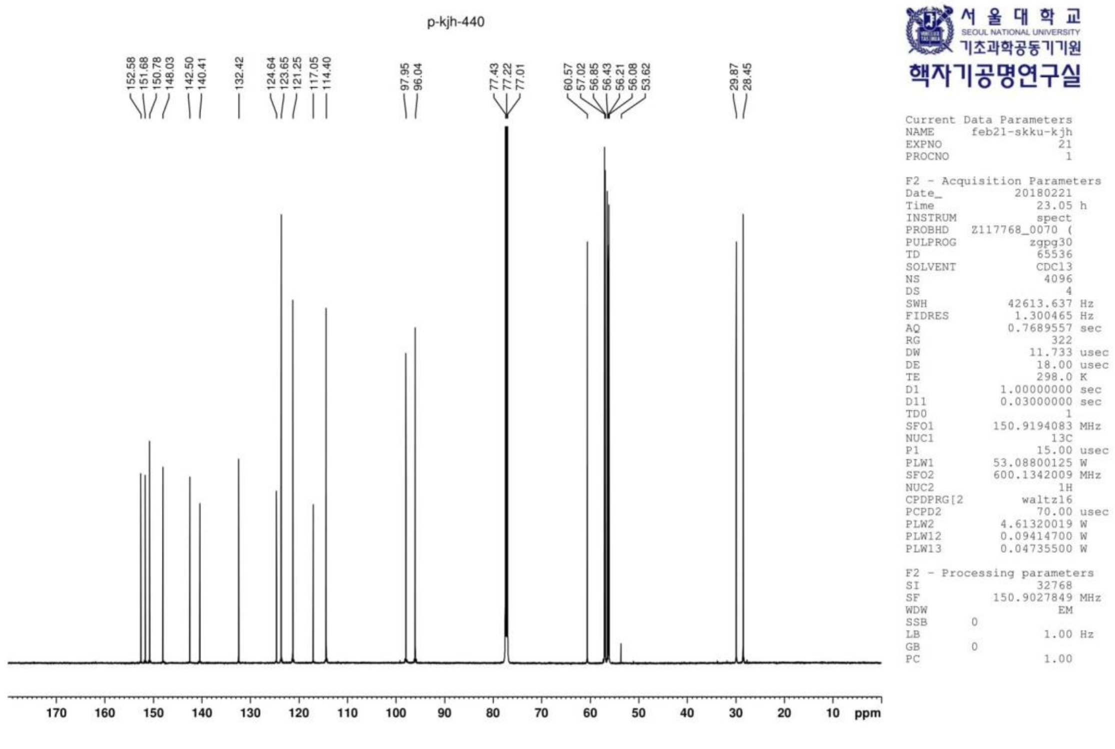

(B)

Figure S3. ^{1}H and ^{13}C NMR spectra of compound **2** ($CDCl_3$, 600 and 150.9 MHz).

(A) ^{1}H NMR spectrum; (B) ^{13}C NMR spectrum

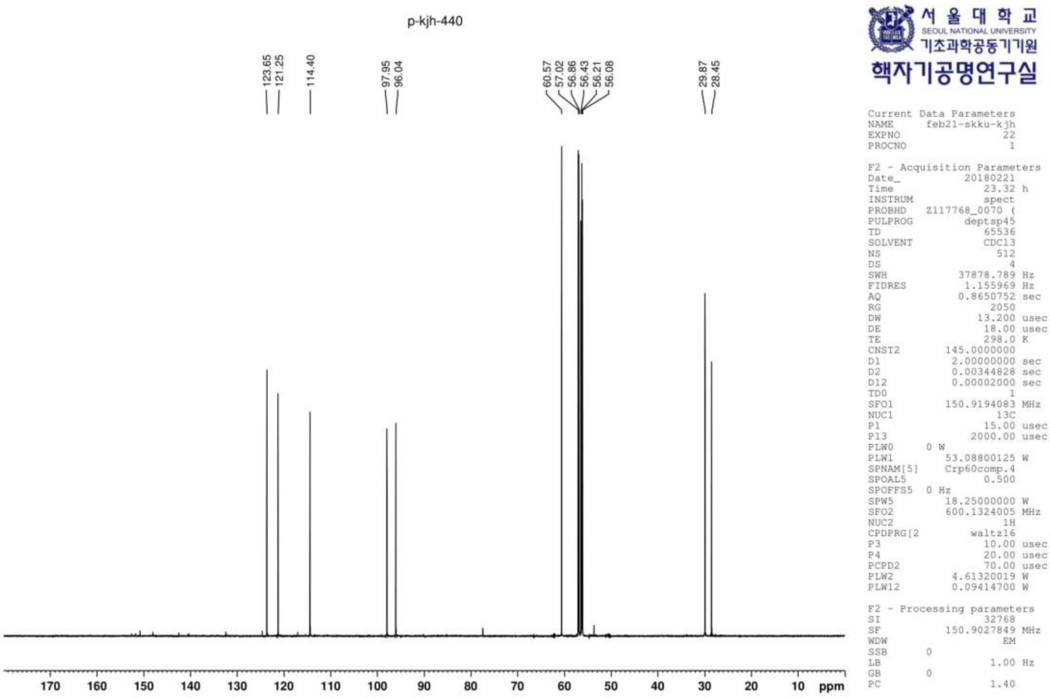
p-kjh-440
123.65
121.25
114.40
97.95
96.04
60.57
57.02
56.86
56.43
56.21
56.08
29.87
28.45
170 160 150 140 130 120 110 100 90 80 70 60 50 40 30 20 10 ppm
서 울 대 학 교
SEOUL NATIONAL UNIVERSITY
기초과학공동기기원
핵자기공명연구실
Current Data Parameters
NAME feb21-skku-kjh
EXPNO 22
PROCNO 1
F2 - Acquisition Parameters
Date_ 20180221
Time 23.32 h
INSTRUM spect
PROBHD Z117768_0070 (
PULPROG deptsp45
TD 65536
SOLVENT CDCl3
NS 512
DS 4
SWH 37878.789 Hz
FIDRES 1.155969 Hz
AQ 0.8650752 sec
RG 2050
DW 13.200 usec
DE 18.00 usec
TE 298.0 K
CNST2 145.0000000
D1 2.00000000 sec
D2 0.00344828 sec
D12 0.00002000 sec
TD0 1
SFO1 150.9194083 MHz
NUC1 13C
P1 15.00 usec
P13 2000.00 usec
PLW0 0 W
PLW1 53.08800125 W
SPNAM[5] Crp60comp.4
SPOAL5 0.500
SPOFFS5 0 Hz
SPW5 18.25000000 W
SFO2 600.1324005 MHz
NUC2 1H
CPDPRG[2 waltz16
P3 10.00 usec
P4 20.00 usec
PCPD2 70.00 usec
PLW2 4.61320019 W
PLW12 0.09414700 W
F2 - Processing parameters
SI 32768
SF 150.9027849 MHz
WDW EM
SSB 0
LB 1.00 Hz
GB 0
PC 1.40

(A)

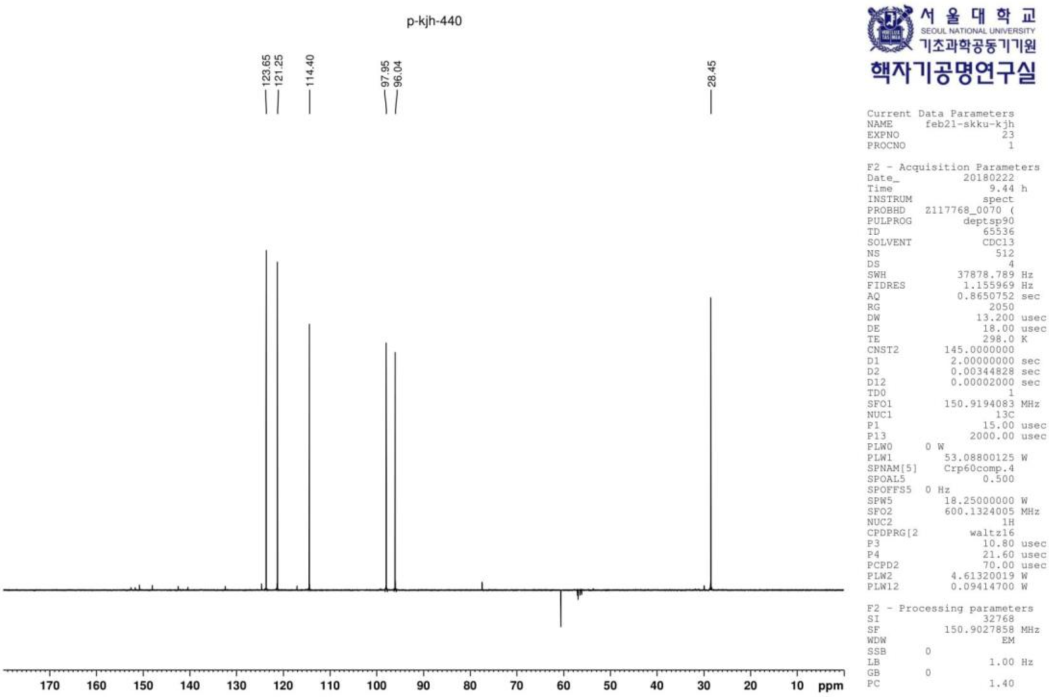
p-kjh-440
123.65
121.25
114.40
97.95
96.04
28.45
170 160 150 140 130 120 110 100 90 80 70 60 50 40 30 20 10 ppm
서 울 대 학 교
SEOUL NATIONAL UNIVERSITY
기초과학공동기기원
핵자기공명연구실
Current Data Parameters
NAME feb21-skku-kjh
EXPNO 23
PROCNO 1
F2 - Acquisition Parameters
Date_ 20180222
Time 9.44 h
INSTRUM spect
PROBHD Z117768_0070 (
PULPROG deptsp90
TD 65536
SOLVENT CDCl3
NS 512
DS 4
SWH 37878.789 Hz
FIDRES 1.155969 Hz
AQ 0.8650752 sec
RG 2050
DW 13.200 usec
DE 18.00 usec
TE 298.0 K
CNST2 145.0000000
D1 2.00000000 sec
D2 0.00344828 sec
D12 0.00002000 sec
TD0 1
SFO1 150.9194083 MHz
NUC1 13C
P1 15.00 usec
P13 2000.00 usec
PLW0 0 W
PLW1 53.08800125 W
SPNAM[5] Crp60comp.4
SPOAL5 0.500
SPOFFS5 0 Hz
SPW5 18.25000000 W
SFO2 600.1324005 MHz
NUC2 1H
CPDPRG[2 waltz16
P3 10.80 usec
P4 21.60 usec
PCPD2 70.00 usec
PLW2 4.61320019 W
PLW12 0.09414700 W
F2 - Processing parameters
SI 32768
SF 150.9027858 MHz
WDW EM
SSB 0
LB 1.00 Hz
GB 0
PC 1.40

(B)

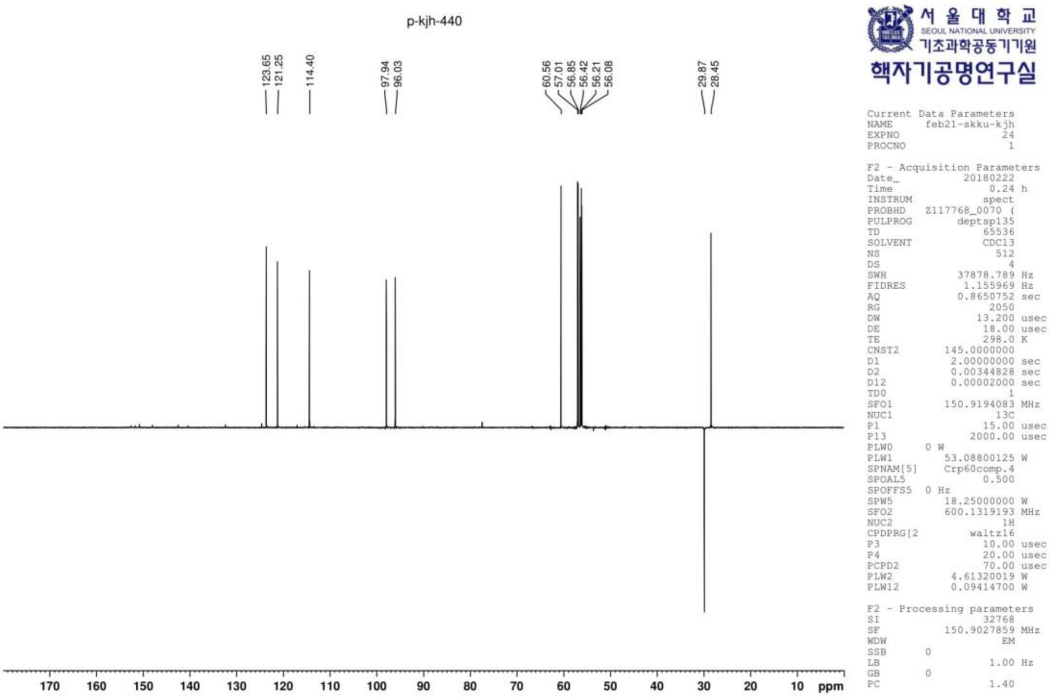

(C)

Figure S4. DEPT spectra of compound **2**

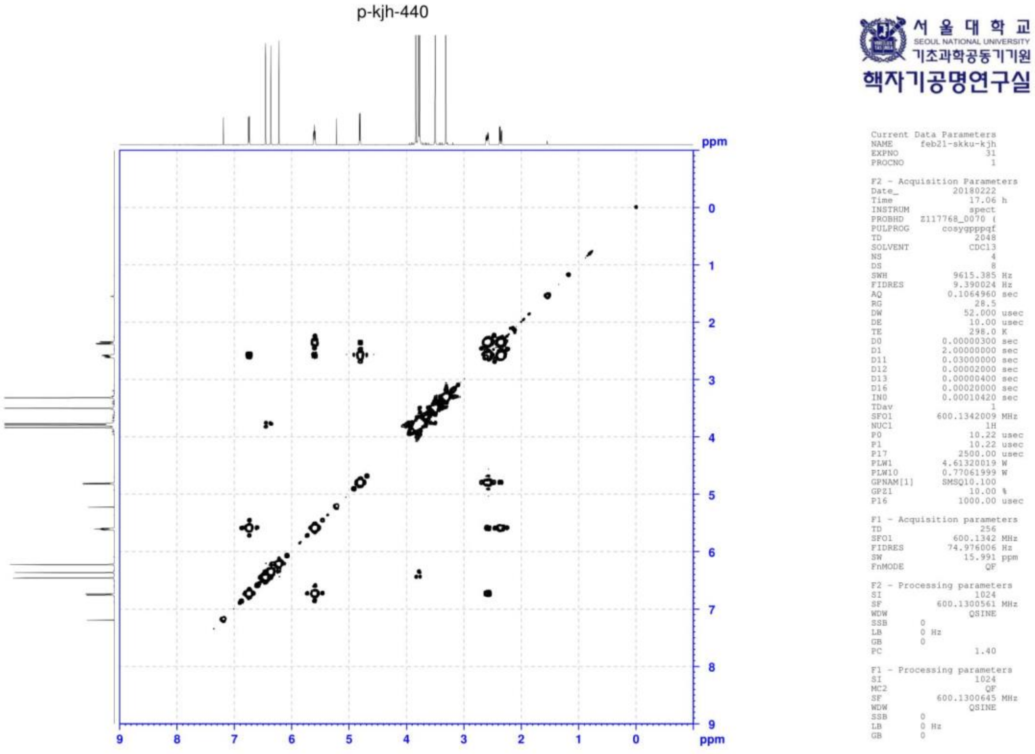

(A)

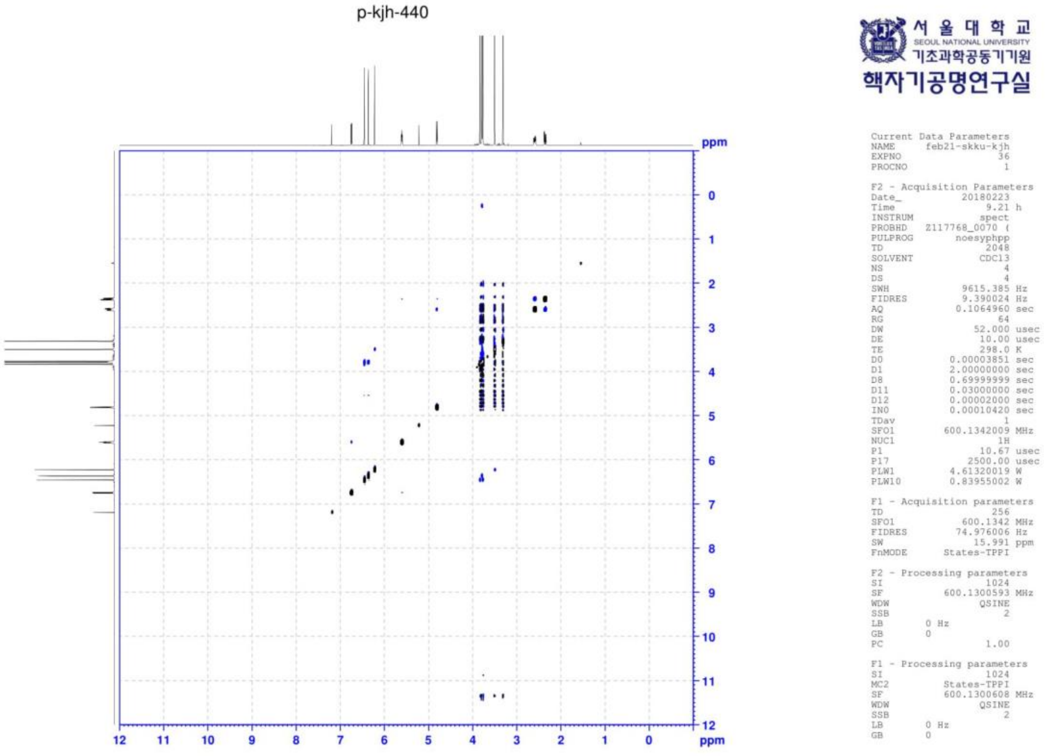

(B)

Figure S5. ^{1}H-^{1}H COSY and NOESY spectra of compound **2**

(A) ^{1}H-^{1}H COSY spectrum; (B) NOESY spectrum

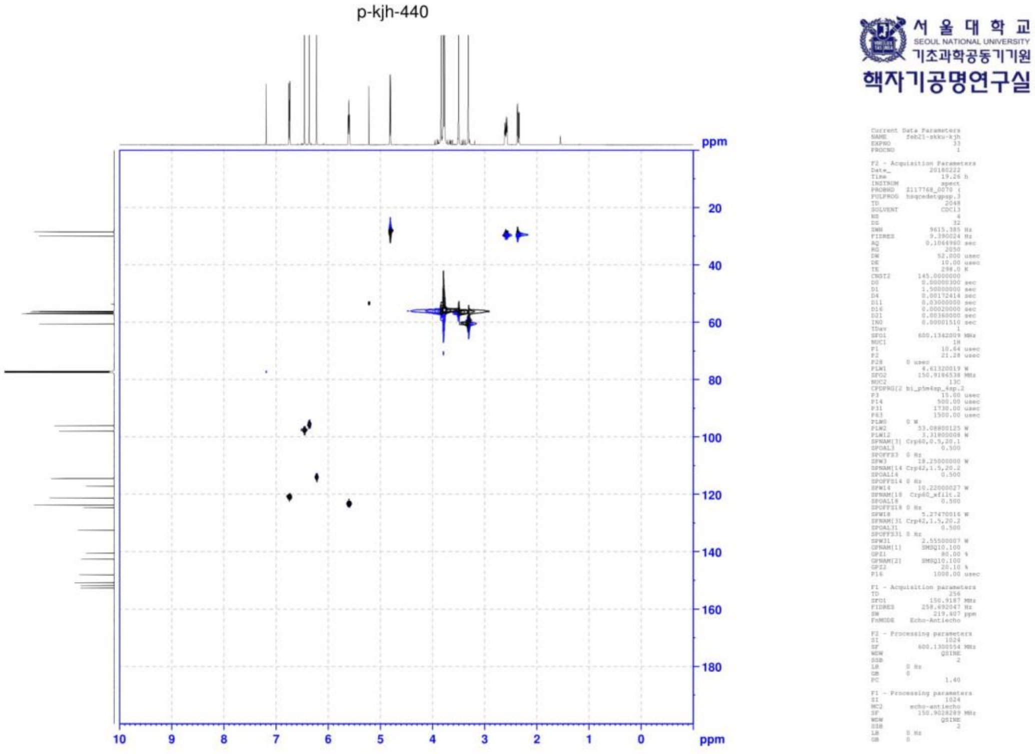

(A)

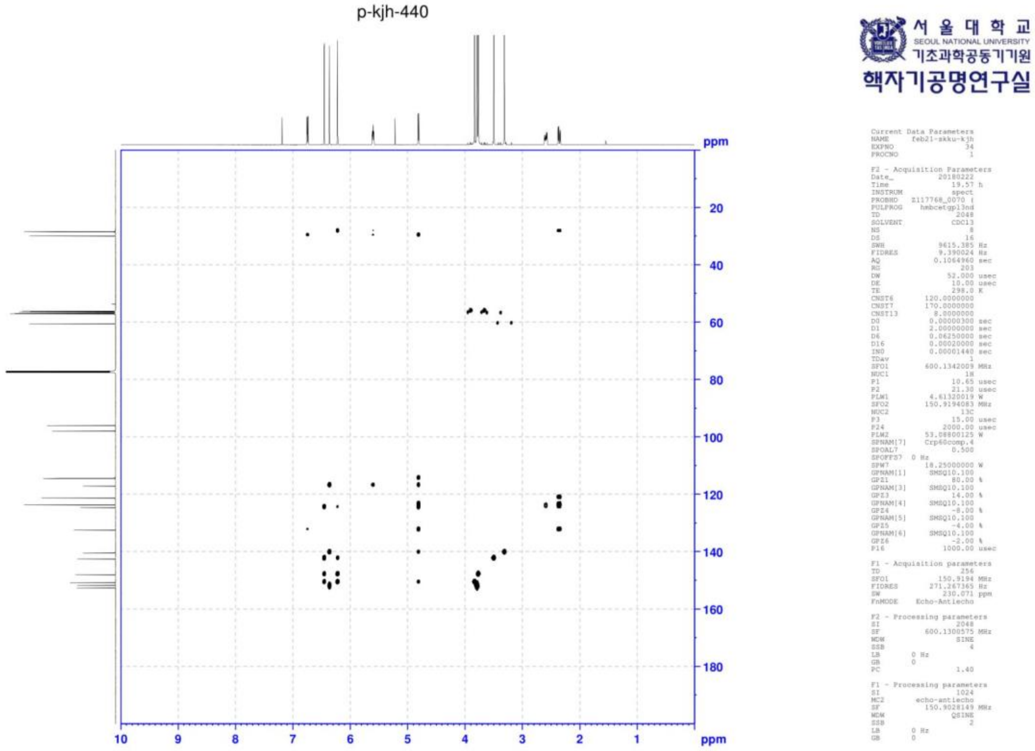

(B)

Figure S6. HSQC and HMBC spectra of compound **2**

(A) HSBC spectrum; (B) HMBC spectrum

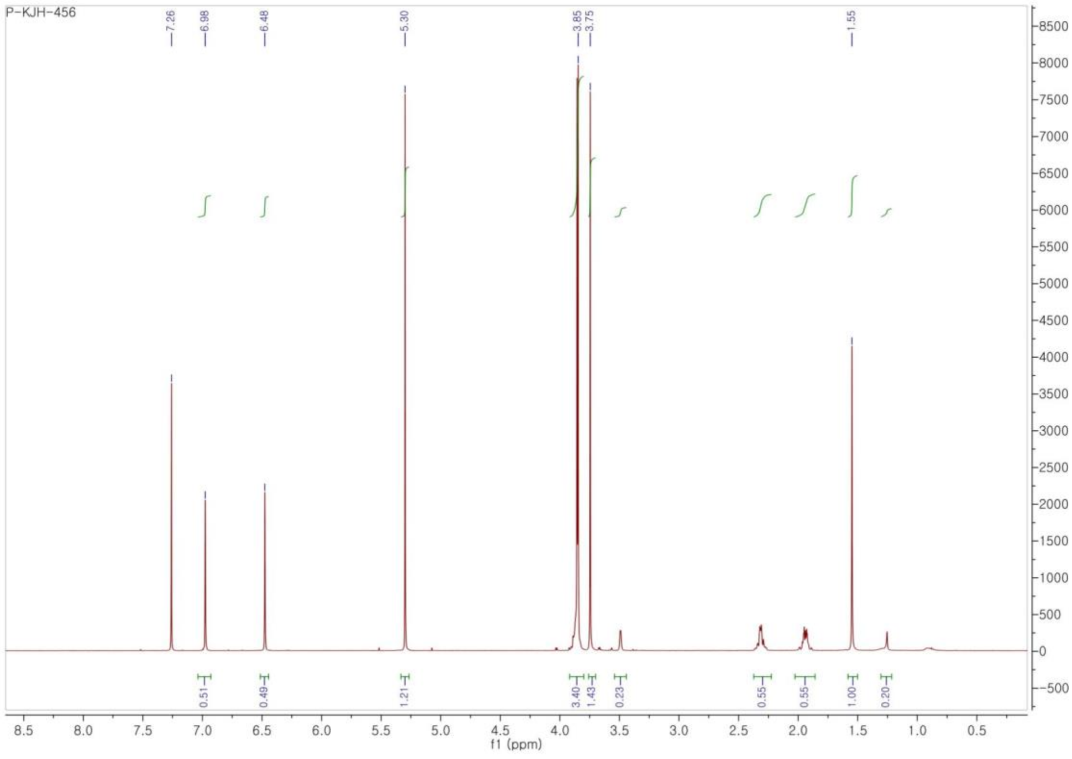

(A)

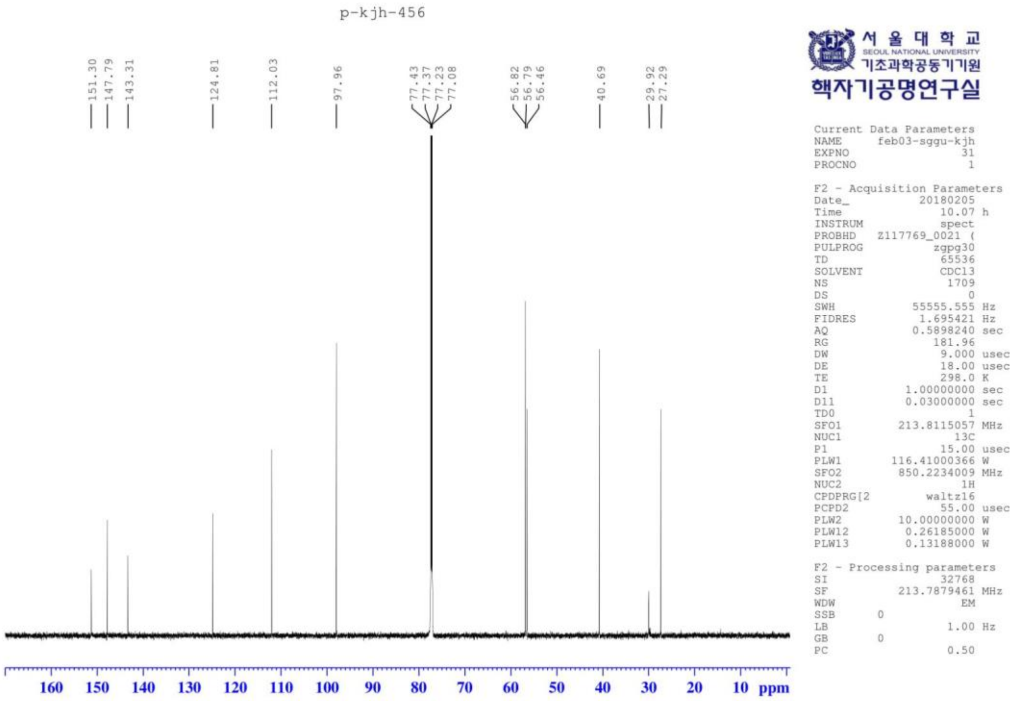

(B)

Figure S7. ^{1}H and ^{13}C NMR spectra of compound **3** ($CDCl_3$, 400 and 213.8 MHz).

(A) ^{1}H NMR spectrum; (B) ^{13}C NMR spectrum.

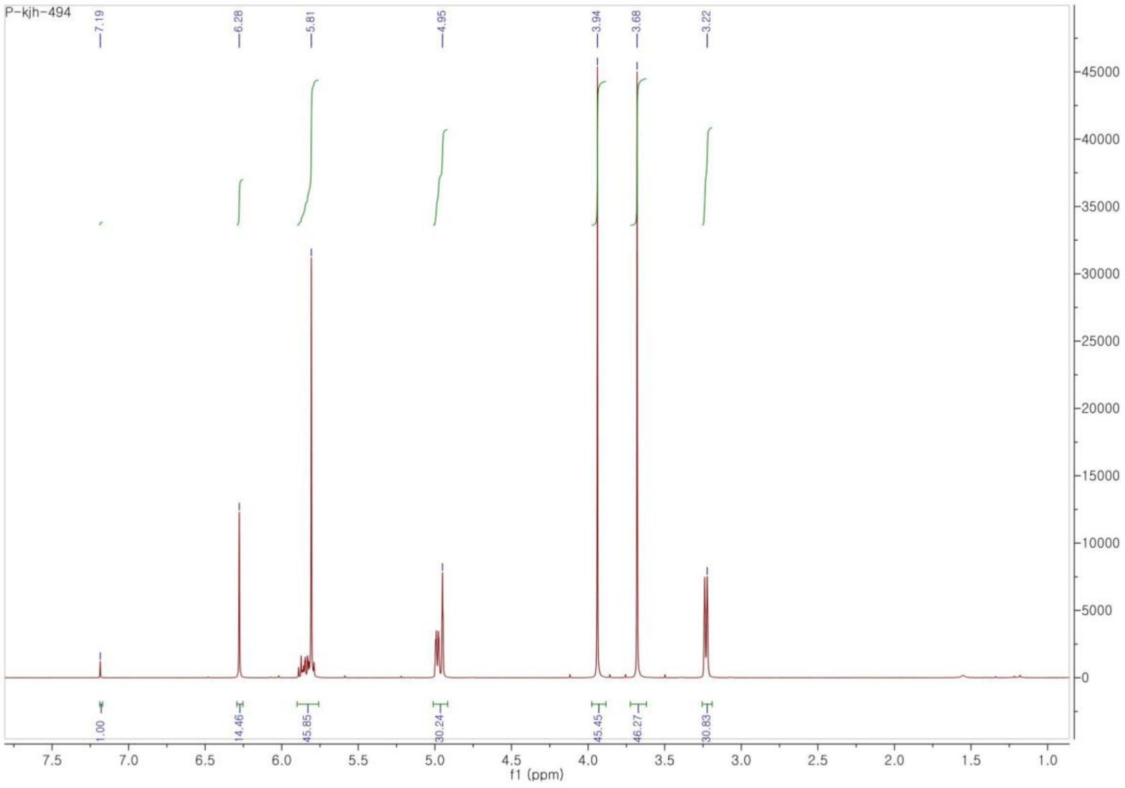

(A)

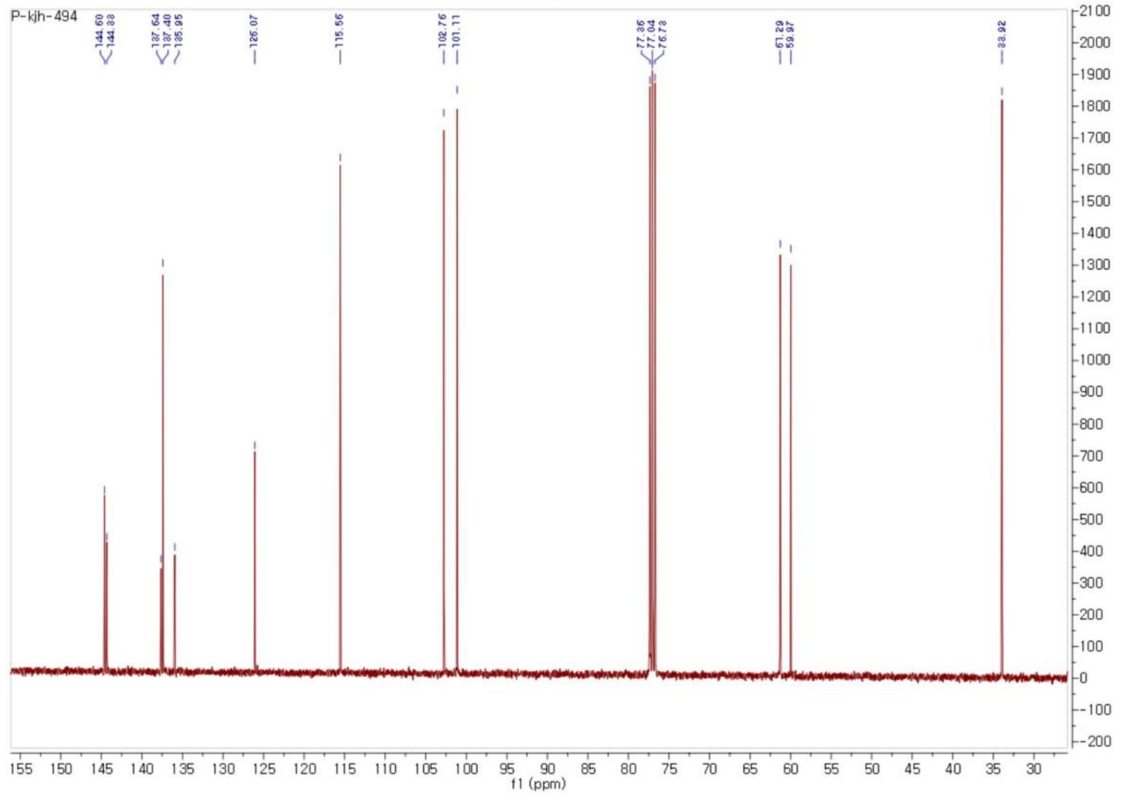

(B)

Figure S8. ^{1}H and ^{13}C NMR spectra of compound **4** ($CDCl_3$, 400 and 100 MHz)

(A) ^{1}H NMR spectrum; (B) ^{13}C NMR spectrum

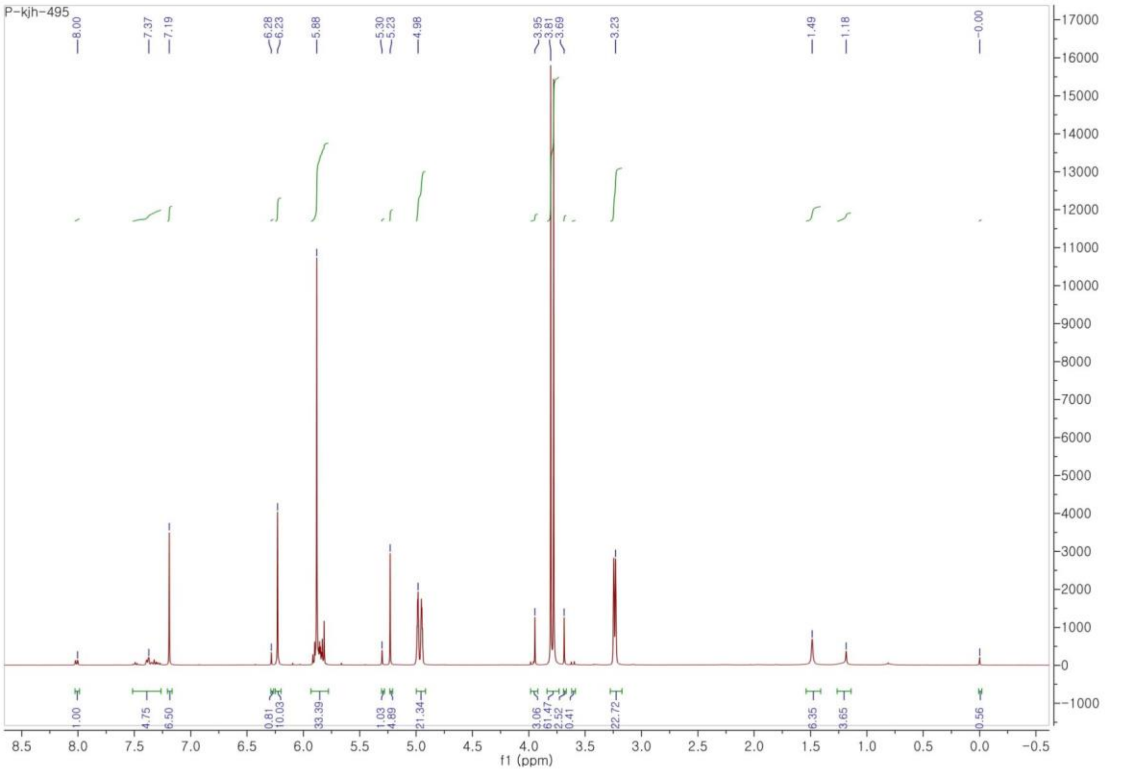

(A)

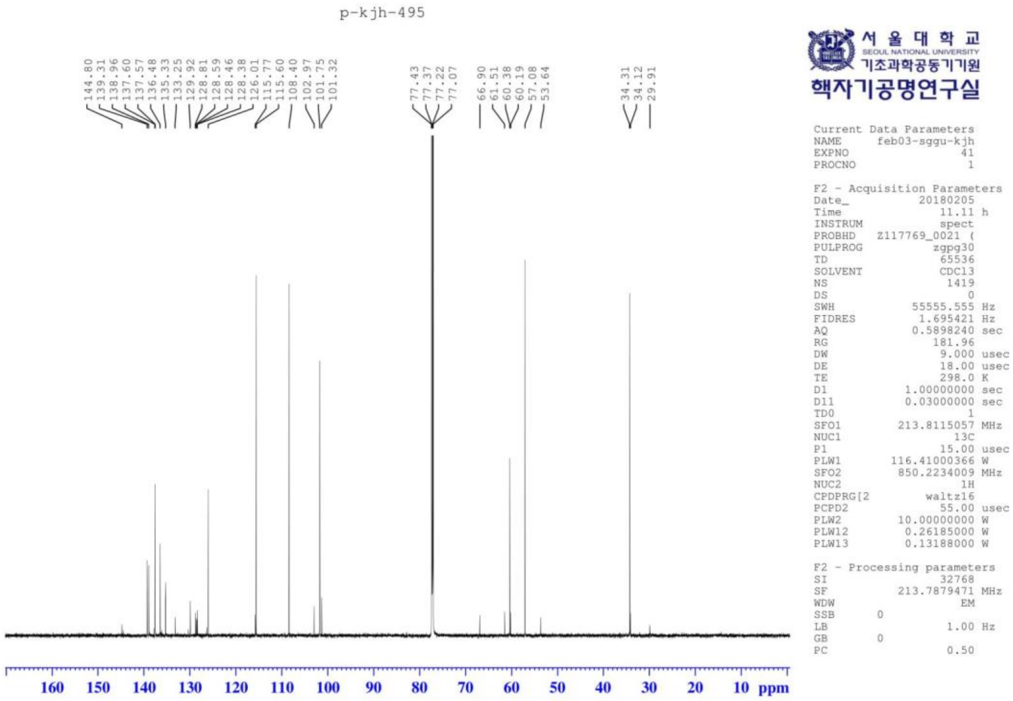

(B)

Figure S9. ^{1}H and ^{13}C NMR spectra of compound 5 ($CDCl_3$, 400 and 213.8 MHz)

(A) ^{1}H NMR spectrum; (B) ^{13}C NMR spectrum

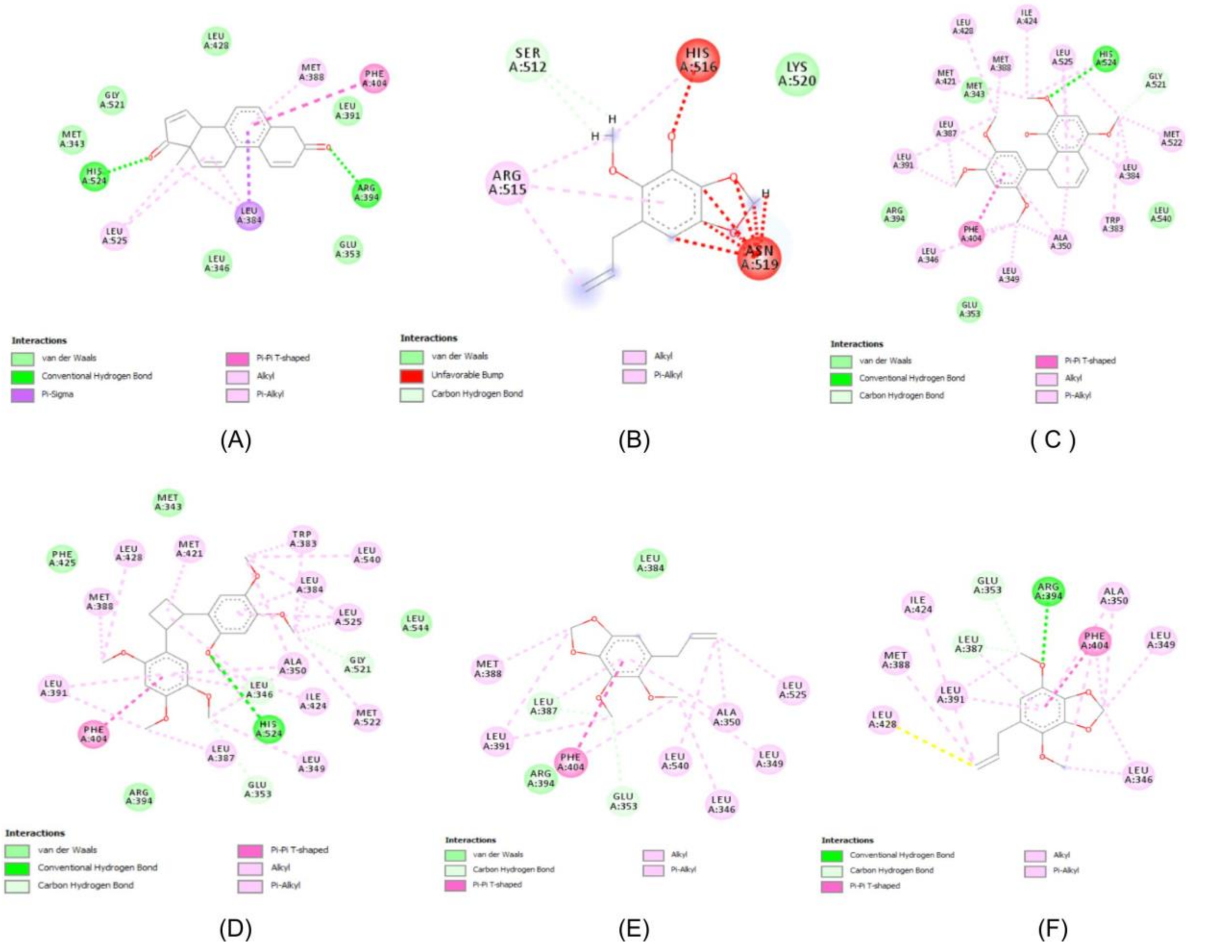

Figure S10. Ligand-Receptor Interaction of Compound **1-5** and Estrogen Receptor α. The figure shown the interaction of estradiol (A), compound **1-5** (B-F, respectively) to estrogen receptor α

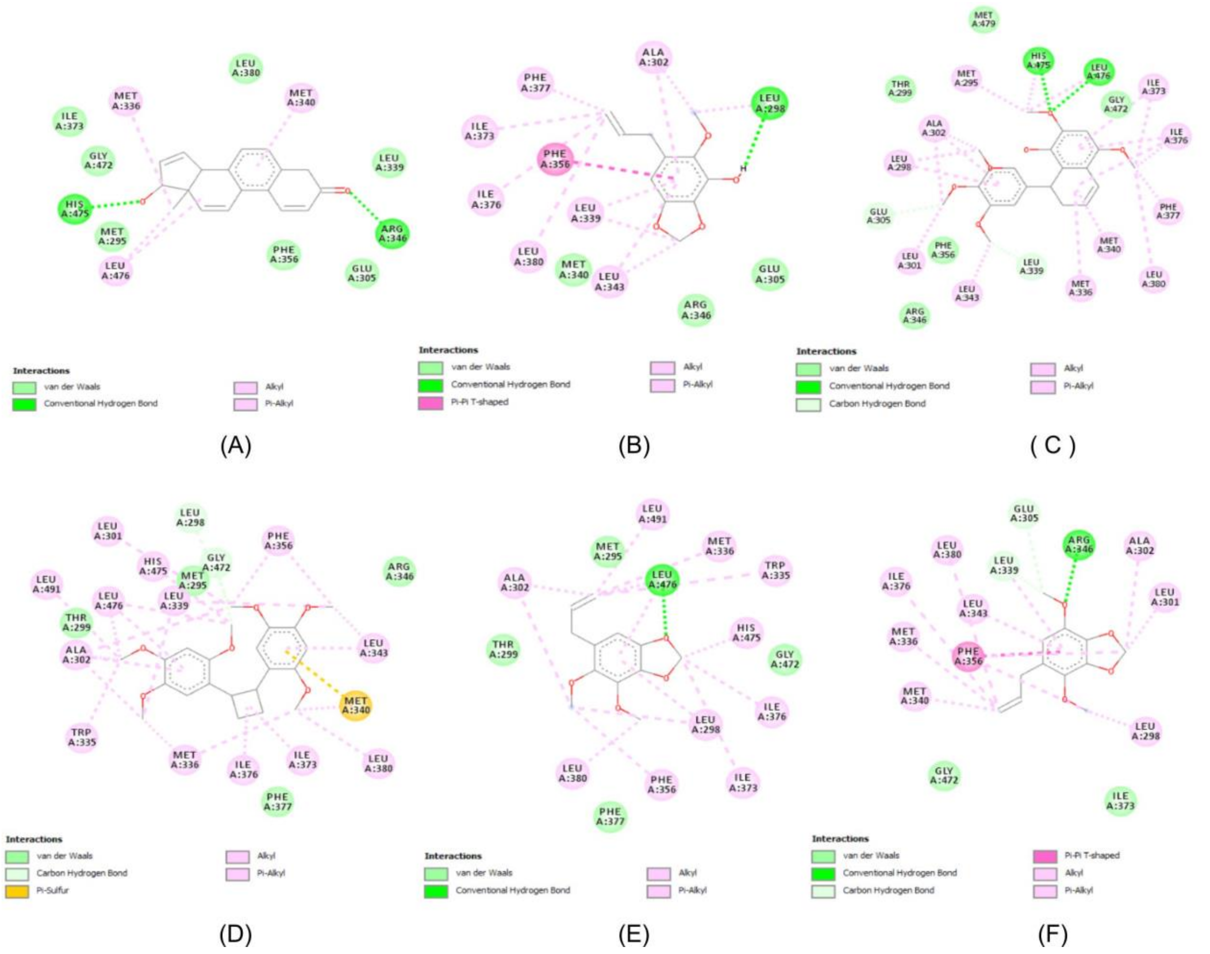

Figure S11. Ligand-Receptor Interaction of Compound **1**-5 and Estrogen Receptor β. The figure shown the interaction of estradiol (A), compound **1**-**5** (B-F, respectively) to estrogen receptor β.
